# Developing and testing a clinical care bundle incorporating caffeine citrate to manage apnoea of prematurity in a resource-constrained setting: a mixed methods clinical feasibility study protocol

**DOI:** 10.1186/s43058-023-00455-x

**Published:** 2023-07-17

**Authors:** Grace Irimu, Ferdinand Okwaro, Jesse Coleman, Mary Waiyego, Florence Murila, Dorothy Chomba, Millicent Parsimei, Cynthia Shitote, Roseline Ochieng, Jasmit Shah, Morris Ogero, Amy Sarah Ginsburg, J. Mark Ansermino, William Macharia

**Affiliations:** 1 Department of Paediatrics and Child Health, University of Nairobi, Nairobi, Kenya; 2 Kenya Medical Research Institute (KEMRI) – Wellcome Trust Research Programme, P.O. Box 43640, Nairobi, 00100 Kenya; 3 Department of Paediatrics, Aga Khan University, Nairobi, Kenya; 4 The University of British Columbia, Vancouver, Canada; 5 Division of Paediatrics, Kenyatta National Hospital, Nairobi, Kenya; 6 London School of Hygiene and Tropical Medicine, London, UK; 7 University of Washington, Seattle, WA USA

**Keywords:** Caffeine, Apnoea, Prematurity, Plan-Do-Act-Study, Quality improvement, Clinical care bundle, Continuous monitoring, Resource limited

## Abstract

**Background:**

Apnoea of prematurity (AOP) is a common condition among preterm infants. Methylxanthines, such as caffeine and aminophylline/theophylline, can help prevent and treat AOP. Due to its physiological benefits and fewer side effects, caffeine citrate is recommended for the prevention and treatment of AOP. However, caffeine citrate is not available in most resource-constrained settings (RCS) due to its high cost. Challenges in RCS using caffeine citrate to prevent AOP include identifying eligible preterm infants where gestational age is not always known and the capability for continuous monitoring of vital signs to readily identify apnoea. We aim to develop an evidence-based care bundle that includes caffeine citrate to prevent and manage AOP in tertiary healthcare facilities in Kenya.

**Methods:**

This protocol details a prospective mixed-methods clinical feasibility study on using caffeine citrate to manage apnoea of prematurity in a single facility tertiary-care newborn unit (NBU) in Nairobi, Kenya. This study will include a 4-month formative research phase followed by the development of an AOP clinical-care-bundle prototype over 2 months. In the subsequent 4 months, implementation and improvement of the clinical-care-bundle prototype will be undertaken. The baseline data will provide contextualised insights on care practices within the NBU that will inform the development of a context-sensitive AOP clinical-care-bundle prototype. The clinical care bundle will be tested and refined further during an implementation phase of the quality improvement initiative using a PDSA framework underpinned by quantitative and qualitative clinical audits and stakeholders’ engagement.

The quantitative component will include all neonates born at gestation age < 34 weeks and any neonate prescribed aminophylline or caffeine citrate admitted to the NBU during the study period.

**Discussion:**

There is a need to develop evidence-based and context-sensitive clinical practice guidelines to standardise and improve the management of AOP in RCS. Concerns requiring resolution in implementing such guidelines include diagnosis of apnoea, optimal timing, dosing and administration of caffeine citrate, standardisation of monitoring devices and alarm limits, and discharge protocols. We aim to provide a feasible standardised clinical care bundle for managing AOP in low and middle-income settings.

**Supplementary Information:**

The online version contains supplementary material available at 10.1186/s43058-023-00455-x.

Contributions to the literature

- A context-sensitive apnoea of prematurity clinical care bundle incorporating the use of caffeine citrate and continuous monitoring of vital signs of all preterm infants born < 34 weeks gestation.
- Identification of critical activities such as clinical audits and stakeholders’ engagement to support the adoption of caffeine citrate for managing apnoea of prematurity.
- Determination of barriers to and facilitators for effective implementation of the apnoea of prematurity clinical care bundle.
- Definition of target quality improvement activities in the pathway for implementing the apnoea of prematurity clinical care bundle.

## Background

Apnoea due to immaturity of the respiratory centre referred to as apnoea of prematurity (AOP), is a common diagnosis in newborn intensive care units in high-income countries. In Kenya, AOP is not among the top diagnosis among preterm infants due to inadequate diagnostic facilities [1]. Incidence of AOP varies with gestational age (GA), with almost all preterm infants with GA < 28 weeks developing AOP, 20% at 34 weeks and < 10% beyond 34 weeks GA [2, 3]. AOP results in hypoxemia and bradycardia that may cause severe long-term disability or death.

Methylxanthines, such as caffeine and aminophylline/theophylline, can help prevent and treat AOP. They work by stimulating the respiratory centre. Caffeine citrate and aminophylline have similar therapeutic effects on respiratory function [4]. However, caffeine citrate is superior to aminophylline in improving the efficacy of supplemental oxygen, less risk of tachycardia and feeding intolerance, more reliable enteral absorption and longer half-life [4, 5]. Recently, the WHO strongly recommended caffeine citrate for the treatment of AOP in preterm infants and the same conditionally, on shared decision-making with the parents, for the prevention of apnoea in preterm infants born before 34 weeks gestation [6]. Caffeine citrate reduces the risk of AOP and its consequences, such as patent ductus arteriosus and bronchopulmonary dysplasia [5, 7, 8]. Additionally, neonatal caffeine therapy is associated with improved visual, motor, perceptual and spatial abilities at the attainment of the age of 11 years by averting adverse effects on intelligence, attention or behaviour, thereby demonstrating long-term safety in very low birthweight neonates [9, 10]. Nevertheless, despite being used widely in high-income countries for prevention and treatment of AOP, caffeine citrate is not available in most resource-constrained settings (RCS) because of its cost limitations [11, 12]. Furthermore, challenges in using caffeine citrate to prevent AOP include identifying eligible preterm infants where GA is not always known and the capability for continuous monitoring of vital signs to identify apnoea. Thus, there is a need to develop evidence-based and context-sensitive clinical practice guidelines (CPGs) to standardise and improve the management of AOP in RCS. Concerns requiring resolution in implementing such CPGs include diagnosis of apnoea, optimal timing, dosing and administration of caffeine citrate, standardisation of monitoring devices and alarm limits, and discharge protocols [7, 13, 14].

We hypothesise that an evidence-based, accessible, simple-to-use and acceptable clinical care bundle incorporating caffeine citrate in the prevention and treatment of AOP will contribute towards best practices in the management of neonates with AOP at a tertiary healthcare facility in Kenya. We aim to develop an evidence-based, context-sensitive prototype clinical care bundle that includes caffeine citrate for the prevention and management of AOP. This will be refined further to improve its feasibility, acceptability, and usability to obtain a scalable care bundle. This will be achieved using a continuous quality improvement strategy that applies a Plan-Do-Study-Act (PDSA) framework integrated with key stakeholders’ engagement, audits and feedback [15, 16].

## Methods

### Study design

This protocol details a prospective mixed-methods clinical feasibility study on using caffeine citrate to manage apnoea of prematurity in a single facility tertiary-care newborn unit (NBU) in Nairobi, Kenya.

This study will start with 4 months of baseline data collection that will focus on the observation of practices related to caffeine use in the management of AOP using qualitative and quantitative approaches. Over 4 months, a clinical care bundle prototype will be developed and improved through PDSA cycles. This will be a pragmatic study and hospital staff will manage all neonates throughout the study period. We will use the following approaches to achieve this: (i) qualitative research in the formative phase, including onsite observations, rapid health facility assessment, in-depth interviews (IDI) and/or focus group discussions (FGD) with key stakeholders, including health care practitioners (HCPs), health care administrators (HCAs) and caregivers, and (ii) quantitative data collection and analysis involving the collection of neonatal patient-level data on the processes of care at baseline. These baseline data will provide contextualised insights on care practices within the NBU that will inform the development of a context-sensitive intervention. The AOP clinical care bundle will be tested and refined further during the implementation phase of the quality improvement initiative using a PDSA framework underpinned by quantitative and qualitative clinical audits and stakeholders’ engagement.

**Fig. 1:**
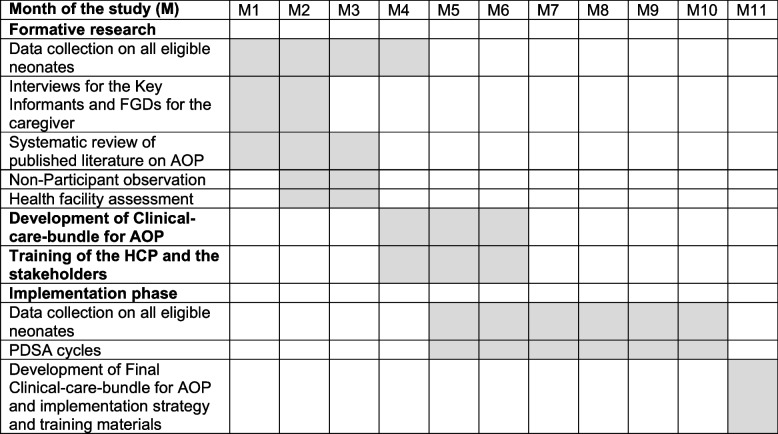
Gantt chart of the research activities in the formative and implementation phase. Abbreviations: AOP- Apnea of prematurity, PDSA- Plan-DO-Study-Act, HCP – Health care practitioners, FDGs-Focused group discussions, NBU- Newborn unit, QI- Quality improvement

Additionally, quantitative data will be collected during the implementation phase that will be utilised to systematically evaluate the adoption of the AOP clinical care bundle (Fig. 1). Qualitative feedback during clinical audits will provide contextualised explanation for the quantitative results. Additionally, the feedback will provide insight on the acceptance and usability, feasibility of scale-up and why and how the clinical care bundle will work or fail to work. PDSA framework was chosen because of its structured experimental approach to testing interventions and allows for adjustment and refinement of the interventions to increase chances of successful delivery and sustainability [15]. The outcome of the study will be a context-sensitive AOP clinical care bundle scalable in other RCS.

### Study site

This study will be conducted at a tertiary care teaching hospital whose NBU admits on average 150 neonates per month. About 13% of the admissions are very low birth weight (1000–1499 g), with 85% admitted on their day of birth (Source: Hospital NBU Database). The NBU has a 60-bed capacity, though average bed occupancy is about 150%, with neonates having to share cots/incubators/radiant warmers. The NBU staffing includes five neonatologists, seven neonatology fellows, one paediatrician, three medical officers and 12–15 paediatric residents. Night coverage is done by one medical officer and one resident. The unit has a complement of about 80 nurses working in shifts, with an average of 13 on each shift. National guidelines for Tertiary Newborn Care are in use at the study site that recommends caffeine citrate for the prevention of AOP for all preterm neonates born at gestational age < 34 weeks. Aminophylline is used as an alternative when caffeine citrate is unavailable.

### Study population and eligibility criteria

The study population for the qualitative study will comprise caregivers, hospital clinicians and support staff. The quantitative data collection will include all neonates admitted to the NBU during the study period (i) GA < 34 weeks and (ii) any neonate prescribed aminophylline or caffeine citrate.

In-depth interviews will be conducted among the healthcare practitioners (e.g. trained nurses, physicians, neonatologists, fellows and residents) in the NBU, and heads of the transdisciplinary units that directly or indirectly offer or influence services delivered in the newborn unit. Additionally, senior hospital leadership, the hospital supplies department and pharmacists involved in procuring, preparing and supplying aminophylline or caffeine citrate will be interviewed. A snowball sampling approach will be utilised to identify other key stakeholders. All stakeholders will be engaged in executing the continuous quality improvement (CQI) during the implementation of AOP clinical care bundles. FGDs will be conducted with the caregivers of neonates enrolled in the quantitative study. All the IDI respondents will be required to have worked in the NBU for over three months preceding this data collection period, while mothers whose babies will have been admitted to the unit for more than 3 days will be considered for inclusion in FGDs.

Although all babies with AOP and those at risk will receive caffeine citrate, only data from babies of caregivers who provide informed written consent will be used in publications from this work. Similarly, FDGs and IDIs will include only those who provide written consent for interviews and data utilisation.

### Sample size

Four FGDs will be conducted with caregivers, each group comprising a maximum of seven respondents. A minimum of 12 and a maximum of 30 HCPs and in-charges of the transdisciplinary units will be enrolled for IDIs. The ultimate number of respondents interviewed will be determined by the achievement of the point of saturation in data collection.

All eligible neonates admitted to the NBU during the formative and implementation phases of the study period will be enrolled in the study.

### Study procedures

#### Baseline data collection for qualitative research in the formative research phase

Data collection methods for the qualitative study will include IDIs, FGDs, onsite non-participants observations and NBU walk-through observation.

*IDIs and FGDs:* The research team will use topic guides for IDIs and FGDs (Additional file 1). IDI respondents will be selected from a list of HCPs at the NBU unit. HCAs will be identified through snowball sampling. HCPs and the leadership of the NBU unit will be asked to identify other hospital personnel involved in the management, provision of care and supplies to the NBU. A social scientist FO and two research assistants (RAs) with experience in qualitative studies will conduct the interviews [17]. The topic guides will be piloted and revised during RAs training. The respondents will be informed by phone call and text messaging 2 weeks before and a day before the interviews. The interviews will be held in a quiet and conducive environment within the hospital to minimise the disruption of the caregivers’ activities. IDIs are expected to last between 45–60 min.

FGDs will be conducted with purposively selected caregivers of neonates enrolled in the study. Each FGD will comprise a heterogeneous group of mothers of varying ages and educational status. Heterogenous groups are targeted to yield rich discussions. FGDs are expected to take between 60 and 90 min.

The IDIs and FGDs will be audio-recorded, and the RAs will document issues such as body language and inordinate silence. Iterative analyses for the IDIs data will be performed with additional stakeholders identified until data saturation is reached. In both IDIs and FGDs, the focus will be on systems rather than on the behaviour of individuals. Thus, this approach will be non-judgmental and recognise that behaviour not consistent with the uptake of best practices could be ‘maladaptation’ to an inefficient health system [18].

*Non-participant observation*: We shall conduct a 2-week non-participant observational research in the NBU. The observations will be guided by a checklist derived from the quantitative questionnaire. The checklist will comprise opportunistic observations of 17 tasks commonly performed in the care of very low birth weight infants in the NBU (Additional file 2). Data collection will require direct observation of care provided to these infants and documentation processes. The results will be evaluated against information documented by the HCPs in the patients’ medical records. Additionally, observations will provide a comprehensive understanding of data collected from the other research methods and provide additional insight into the implementation context.

Two study nurses MP and CS will conduct the observations. They will be trained on the best practices for clinical tasks using the clinical procedure guidelines used in training the NBU staff. The study nurses will be responsible for quantitative data collection, a 3-month period during which they will have developed an insider ‘emic’ perspective despite not being directly involved in routine nursing work. The study nurses will observe independently, each following a different opportunistic task. No HCP will be observed more than once for the same task to avoid data duplication. The observers will remain at a discrete distance and will not engage those observed in verbal or nonverbal communication. The observers will be detached from their professional roles as nurses during the entire study period, allowing them to be objective without being judgmental. The observers will use stopwatches, guided by the checklist, to document the duration of activities. The observers will immediately fill out the checklist in Research Electronic Data Capture (REDCap) tool and enter other observations in ‘shorthand free text’ to avoid recall bias. The observers will report what they observe without interpreting. The investigators, led by an implementation science-trained researcher (GI) with experience in participant observation, will perform regular reviews of the data collected and check on the reflexivity of the observers [19, 20].

*NBU walk-through observation*: A health facility assessment (HFA) tool to evaluate the hospital readiness to implement the clinical care bundle of AOP incorporating caffeine citrate and continuous monitoring of vital signs will be developed. The tool will be adapted from the WHO Integrated Quality of Hospital Care Assessment and Improvement Tool for Maternal and Neonatal Health (unpublished). Data collection methods will include a walk-through observation and interviews with the nurses and clinicians. Data will be collected on hospital health statistics, the structure of the NBU, the hospital support system, staffing and capacity building, essential drugs, equipment, supplies and laboratory support and other aspects of care not captured in the quantitative data.

#### Baseline data collection for quantitative research

##### Enrollment of neonates

Caregivers of the eligible neonates will be introduced to the study by the study nurses, who will, in turn, obtain written informed consent from each caregiver (Additional file 3). The consent obtained will be on caregivers’ approval for the research team to collect and use anonymised routine patient data for research purposes. Subsequently, neonates will be assigned study participant identification numbers.

##### Data collection

Anonymised participant data will be collected in study logs and digital data collection forms that will be managed using REDCap tools (Additional file 4) hosted at Aga Khan University (AKU) [21, 22]. Patient data documented at admission and during the NBU stay will be extracted manually from hospital’s structured paper record forms. Thus, data on neonatal biomedical data (GA, sex, weight), clinical assessment, comorbidities, mode of delivery, Apgar score and any resuscitation methods at birth will be abstracted from the patient’s admission notes. The following information will be collected daily from a comprehensive newborn monitoring chart (default), or nursing cardex, daily doctors’ notes and referral notes: (i) infant’s weight; (ii) frequency of vital signs documentation, the highest and lowest heart rate and respiratory rate, lowest body temperature and peripheral oxygen saturation (SpO_2)_) for each day; and (iii) number and frequency of apneic episodes (if any). Information to be abstracted daily from patients’ medical records include (a) new diagnoses; (b) mode of respiratory support (if any) and medication data, including caffeine citrate or aminophylline dosing (dose and frequency), timing and treatment duration; and (c) interventions (e.g. kangaroo mother care, intervention for any apnoea including stimulation, airway repositioning, bag and mask ventilation and investigations to establishing secondary causes of apnoea such as spot body temperature, blood sugar and blood culture). Enrolled neonates will be followed for 7 days after discontinuing caffeine citrate or aminophylline treatment or up to 35 weeks post-menstrual age, whichever occurs later. Data for neonates who undergo escalation of care to CPAP or mechanical ventilation will continue to be collected.

Enrollment of eligible neonates and patient-level data collection in the quantitative research will be conducted by two study nurses (MP, CS) who are Good Clinical Practice (GCP) certified. They will follow a standard operating procedure (SOPs) manual that will guide their training.

#### Establishing a collaboration with the hospital staff in research activities

The target activities that will require the hospital staff’s participation include developing the context-sensitive clinical care bundle, identifying the quality indicators to monitor the implementation of the bundles and engaging in clinical audit cycles. The first step will be for the research team to share the preliminary report of the baseline quantitative and qualitative data with the hospital team. The quantitative data will be summarised in tables to demonstrate the biomedical and clinical characteristics of the neonatal study population, frequency and measurements of vital signs and documentation of apnoea episodes. The quantitative data will be explained using qualitative data. The qualitative data and the health facility assessment will be summarised into ‘strength’ in the current practices as depicted by the quantitative data while identifying areas that ‘offer opportunities for improvement’ and the implication to the development of the clinical care bundles and training needs. This will provide insight to and reflection on the current practices in the prevention and management of AOP and provide insight into the improvement in service delivery that would support the adoption of the intervention. Areas that need improvement before implementing the clinical care bundles will be identified and discussed with the hospital team.

#### Development and training on application of clinical care bundles

The research team will develop the first draft of clinical care bundles for AOP incorporating caffeine citrate and continuous monitoring of all preterm infants at risk of AOP guided by existing scientific evidence and reflections of the formative research. The bundle will include (i) a context-relevant evidence-based algorithm to guide the identification of high-risk preterm infants, prescription of caffeine citrate and monitoring of the occurrence of apneic episodes; (ii) a guide on oral and intravenous caffeine citrate administration; (iii) step-by-step instructions on the use of the continuous monitoring devices, including setting alarms; and (iv) evidence-based training materials for dissemination of the clinical care bundle. Other job aides may be developed to address procedural knowledge needs identified in the formative research. These items will be developed through collaboration between the researchers and hospital teams. The clinical care bundle will be context-specific by incorporating the formative phase’s preliminary results and the local experts’ opinions (neonatologists and neonatal nurses within and outside the study site).

**Fig. 2:**
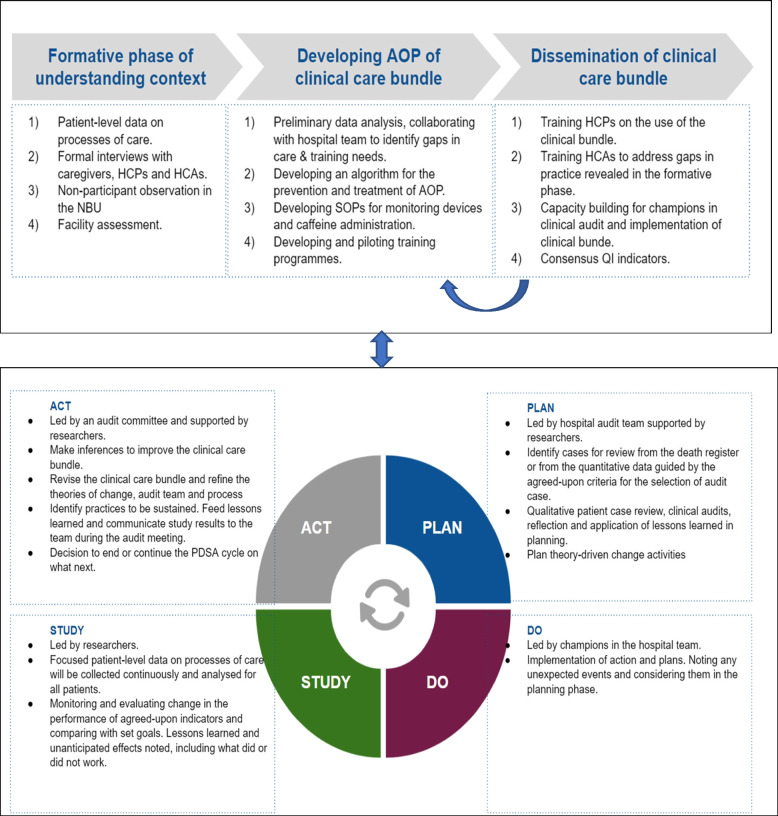
Developing and disseminating a context-sensitive clinical care bundles and testing the bundle in real work setting using the Plan-Do-Study-Act cycles in an implementation phase. Abbreviations: AOP, apnoea of prematurity; PDSA, Plan-DO-Study-Act; HCA, health care administrators; HCP, health care practitioners; NBU, newborn unit; QI, quality improvement; SOP, standard operating procedures

Training materials will be developed for dissemination of the intervention in the form of sessions comprising plenary lectures, scenarios, simulations, and small group practical sessions to provide hands-on experience in performing the corresponding clinical procedures using job aides, dummies and monitoring devices. We shall conduct 1-day training and aim at training at least 60% of all the nurses and 90% of the paediatric residents rotating in NBU during the study period. We will count on the trained to pass on the knowledge and skills to the rest of the NBU staff through on-job training supported as needed by the study neonatologists and nurses and champions that will be identified during the training. An immediate outcome of the training will be a prototype of the AOP clinical care bundle and a consensus on key performance tracer indicators that will monitor the adoption of the clinical care bundle in the implementation phase. Additionally, interactions during the training will be central in identifying potential early adopters and “champions” to enlist in the implementation phase (Fig. 2).

#### Implementation phase (Fig. 2)

##### Qualitative research procedures during implementation phase

A continuous quality improvement (CQI) approach that utilises a theoretical framework based on the Berwick Rules will be applied in the implementation of the prototype AOP clinical care bundle [23]. Multiple cycles of QI following a PDSA framework will be performed for 4 months, ensuring that the clinical care bundle is tested, adapted to context and accepted by users [24] (Table 1).

**Table 1:** Key metrics for the adoption of and adherence to monitoring neonates and managing apnoea of prematurity (AOP) with caffeine citrate

**Description of metrics to be measured key quantitatively measurable outcomes**
a. **Adoption of neonate monitoring devices**
i. Percentage of eligible neonates connected for continuous and intermittent vital signs monitoring
ii. Percentage of neonates at risk of AOP connected to continuous/intermittent vital signs monitor
iii. Duration between neonates’ first dose of caffeine citrate and connection to continuous vital signs monitor
iv. Duration monitored while on caffeine and on discontinuation where applicable (beyond would be for other reasons)
v. Alarm triggered, response times, type of intervention, e.g. escalation of care/stimulation, cardiopulmonary resuscitation, etc
b. **Adherence to monitoring neonates**
i. The percentage of time neonates are connected to continuous vital signs monitoring while at NBU
ii. Average Perfusion index (Pi) as per vital sign monitoring device
iii. Number of customised alarm limits (high/low HR, RRp, temp and low SpO_2_) per neonate
iv. Number of alarms triggered by the device (for each vital sign, and total)—clinician response time and intervention
v. Length of time (in seconds) between alarm triggering and alarm is silenced or cancelled via vital signs monitor (for each vital sign, and total)
c. **Adoption of managing AOP with caffeine citrate**
i. Percentage of neonates at risk of AOP receiving caffeine citrate
d. **Adherence to managing AOP with caffeine citrate**
i. Did the neonate have a new or ongoing caffeine citrate or aminophylline prescription during the 24 time period of interest?
ii. Number of doses of caffeine citrate given in the previous 24-h period
iii. Were caffeine citrate/aminophylline doses missed?
iv. Why were doses missed?
v. Adverse events and comorbidity
**Note**: Sub-group analysis by each room in the New-born Unit depending on the intensity of care required for all above metrics will be performed
**Description of metrics to be assessed qualitatively**
1. Adaptability: ability to adapt the apnoea of prematurity clinical care bundle to local needs
2. Appropriateness: alignment of the apnoea of prematurity clinical care bundle with organisational and individual values, mission, and priorities
3. Complexity: degree of difficulty or effort in administering different components of the apnoea of prematurity clinical care bundle. Identify how difficult various clinicians feel it is to administer the apnoea of prematurity clinical care bundle
4. Culture: political, economic, or institutional norms, values, or assumptions influencing the adoption of the apnoea of prematurity clinical care bundle
5. Acceptability: stakeholder satisfaction with the apnoea of prematurity clinical care bundle

*AOP* Apnea of prematurity, *NBU* Newborn Unit, *HR* Heart rate, *RRp* plethysmographic respiration rate measurement, *SpO*_*2*_ Oxygen saturation

*Audit team and committee to support the PDSA cycles:* The NBU unit has ongoing audits carried out once a month. The investigators will introduce the hospital’s audit team to WHO guidance on case/mortality reviews, conducting facility-based audits, problem framing and root-cause analysis, solution identification and system thinking [25, 26]. Audit processes such as frequency of meetings, duration, venue and selection criteria of neonates for qualitative case reviews will be determined in collaboration with the existing hospital’s audit team. Additional audit team members may be considered as per the WHO guidance. The audit teams will be required to attend the meeting and participate in planning for change, identifying problems and solutions and implementing the planned actions. The audit processes will be tailored to the context, case-mix and performance of tracer indicators.

The NBU and hospital leadership will be engaged to identify an audit committee comprising the in-charges of the transdisciplinary units that are likely to influence the adoption of the clinical care bundles directly or indirectly, such as senior hospital leadership, pharmacists, medicines and equipment procurement and supplies departments. The audit committee members will be expected to join the audit meetings.

*The CQI process using audit feedback and PDSA cycles*: The key focus will be a CQI approach that will identify and overcome barriers to or encourage facilitators to support adoption of the clinical care bundle that can inform a larger-scale multi-site intervention. A PDSA framework, audit feedback and stakeholders’ engagement will be used to promote the adoption of the prototype AOP clinical care bundle. We plan to primarily use the Capability, Opportunity, and Motivation model of behavior change (COM-B) to strengthen the health system to support change by building on the facilitators for and mitigating potential barriers to adopting the clinical care bundle [27]. The PDSA framework will have four steps: (i) Planning for change, (ii) Do—implementing change, (iii) Study—monitoring the success of the adoption of the clinical care bundle and (iv) Act—adapting the clinical care bundle and the implementation process to the context [15, 16] (Fig. 2).

*Planning for change:* Designated team members will prepare case reviews to be presented during the audit meeting. A neonatal audit tool used at the NBU over the previous 2 years will be used for data collection and guide to the case presentation. The audit team members will identify inconsistencies in neonate assessment, indications and prescription of caffeine citrate, monitoring and identifying modifiable factors in the care processes. We shall use the term ‘modifiable’ instead of ‘avoidable’ because the former limits opportunities for blame and indicates the potential for positive change [25]. Recommendations in the clinical care bundles will be used as the audit criteria. The root cause analysis will be performed to identify problems and potential solutions, as well as to understand ‘what works’, for whom, and facilitators of and barriers to adopting the AOP clinical care bundle. The practice to change, the level at which change should occur, the person(s) responsible, the resources required and the timelines will be agreed upon during the audit meetings [25].

At the beginning of every audit meeting, the participants will sign a non-disclosure clause that prohibits discussions of the proceedings of the clinical meeting outside of the group.

*Do:* The hospital staff will implement the solutions identified.

*Studying:* Regular audit reports on the performance of the tracer indicators will be prepared using individual patient-level data collected in the quantitative research during the implementation phase (see below).

*Act by adapting the clinical care bundle or implementation processes:* The audit committee will make inferences from the performance of the tracer indicators and deliberate any revision of the clinical care bundle and the implementation process, including theories of change, the composition of the audit team and reflect on the learning experiences. The decision to end or continue the PDSA cycle will be made by this committee as well the decision of ‘what next’ (Fig. 2). The actions of the audit committee will be communicated to the audit team in the subsequent meeting.

PDSA processes will be repeated to refine the AOP clinical care bundle to enhance its adoption and scalability (Fig. 2). The frequency and number of PDSA cycles will depend on the severity and magnitude of the modifiable factors. The key focus will be to have CQI that will identify and overcome barriers to and/or encourage facilitators for adoption that can be used to inform a larger-scale multi-site intervention. The study neonatologists MW and FM, based on the study NBU, will lead the CQI processes. They will be supported by an implementation research scientist (GI) with experience in CQI and root cause analysis in the complex social systems of health care [18, 25, 28]. Satisfaction regarding the AOP clinical care bundle will be explored during these multidisciplinary audit meetings.

##### Quantitative data collection during implementation phase

The identification of eligible neonates, informed consent process and neonate enrolment during the implementation phase is similar to processes described for baseline data collection in the formative research phase. All neonates will receive care per hospital policies at the discretion of the hospital team. However, the admitting clinician will be encouraged to perform the New Ballard Scoring for GA assessment and the Silverman-Anderson respiratory severity scoring at the admission of neonates. Additionally, the clinicians will be encouraged to prescribe caffeine citrate to prevent (or treat) AOP and instruct continuous monitoring of vital signs for all neonates < 34 weeks GA as per the clinical-care-bundle recommendations. A multi-parameter vital sign monitoring device will be connected to the patient by the hospital staff following the SOPs developed as part of the clinical care bundle. The device can digitally record the heart rate and oxygen saturation and provide user-adjustable alerts for notification and documentation. The data obtained from the monitoring device will supplement the clinical data extracted from the neonate’s admission notes and treatment charts as described in the formative research phase. Continuous monitoring of the vital signs will be performed for at least 7 days after administration of the last dose of caffeine citrate or until the neonate is discharged from the NBU as per hospital guidelines.

Patient data will be captured, stored and tracked in the REDCap data collection tool by study staff, whose training will be informed by the SOPs for this study. The hospital staff will be trained on patient assessment of the parameters in the data collection tool.

*Follow-up period of all neonates included in the study:* All neonates on caffeine citrate or aminophylline for prevention or treatment AOP will be followed for 7 days after discontinuation of caffeine citrate or aminophylline by the primary physician or up to 35 weeks GA, whichever occurs later. Follow-up will be continued even when the level of care is escalated (e.g. to CPAP or mechanical ventilation). We recognise that simply having an AOP clinical care bundle and implementation strategy does not lead to change; rather, attention and effort will be required to have a system that supports change by building on the facilitators for and mitigating potential barriers to adoption of the bundle. We plan to primarily use the Capability, Opportunity, and Motivation model of behaviour change [29].

#### Withdrawal and early termination

Neonates and their caregivers may decline care given by the hospital staff or voluntarily withdraw from the study for any other reason at any time. The HCPs may also withdraw neonates from the study to protect their safety if, in their opinion, continuing participation would jeopardise the neonate’s health. HCPs, HCAs and caregivers may voluntarily withdraw from the qualitative arm of the study for any reason at any time. The appropriate study forms will document any participant withdrawal or early termination.

### Data analysis

#### Qualitative data analysis

Qualitative data in narrative format will be analysed to assess the feasibility, usability and acceptability of monitoring neonates and managing AOP with caffeine citrate among HCPs and HCAs, and acceptability among caregivers. The findings will be presented in a descriptive narrative. The field notes for non-participants’ observation will be coded and analysed using a codebook with identified themes, including the barriers to, facilitators of and feasibility of using monitoring technologies and caffeine citrate, perceived value and training needs. Qualitative data analysis software will be used to organise, code and analyse the qualitative data in an iterative process. The research team will identify an initial set of codes and themes based on the categories of the qualitative data, adding any emergent issues and themes. Any discrepancies in coding will be discussed and resolved. Observations and field notes will augment and contextualise IDI and/or FGD data.

#### Quantitative data analysis plan

Summary statistics will be presented as frequencies and proportions for categorical data and as means and standard deviations or medians and interquartile ranges (where appropriate) for continuous data. Where applicable, 95% confidence intervals will be stated. The normality for the continuous data will be analysed using the Shapiro–Wilk test. Regression analysis will be performed when determining associations with the risk factors. Chi-squared test or Fisher’s exact test will be performed on categorical data when comparing two or more groups. Student’s *t*-test or Mann–Whitney *U* test will be performed on continuous data when comparing two or more groups. For visualisation purposes, bar chats, density plots and time series plots will be used for variables where appropriate. A *p*-value < 0.05 will be considered statistically significant (Table 1).

## Researchers’ requirements

All researchers will have valid certification in the Good Clinical Practice (GCP) prior to any interactions with study participants. Prior to the commencement of this study, all researchers will have clearly defined study roles and will receive study-specific training on any study tasks or procedures necessary to carry out their responsibilities. These include the training on the study protocol, SOPs, data collection tools, informed consent process, conducting clinical audits including root cause analysis and understanding of the theories applied in this study. Training will be conducted by the study team or their designee as appropriate. All researchers will hold weekly conference calls to check the study progress, any significant events in the study site and any protocol adaptation or deviation and action taken.

Additionally, the local researchers will meet virtually or physically once a week to discuss study procedures and ensure that the research team will implement the research as outlined in the protocol. This is a pragmatic study; thus, it will not be conducted in a controlled setting. A careful account of any adaptation in any component of the intervention or implementation strategies and any changes in the context in response to barriers and contextual factors will be made [30].

### Additional resources to support to enhance the adoption of clinical care bundles of AOP in the study site

The research team will ensure that the study hospital has an adequate supply of caffeine citrate and that all eligible neonates receive AOP prevention or treatment during the implementation phase. Further, the research team will provide the hospital with multi-parameter vital sign monitors to allow continuous vital sign monitoring for all patients born at less than 34 weeks GA. This monitoring device is approved for neonates and is commercially available in Kenya.

## Ethics consideration

The study described in this proposal has been approved by the Kenyatta National Hospital/University of Nairobi Ethics Review Committee (P922/11/2021) and the Aga Khan University Nairobi Ethics Committee 2019/IERC-165 (V1) (Nairobi, Kenya) (Additional file 5 and Additional file 6). Any future modifications to this protocol will require approval from these Review Committees.

All engaged stakeholders will have provided written informed consent. No identifying information will be retained for any neonate whose caregiver declines study participation. Data of neonates whose caregivers decline to participate in the qualitative study interviews will be included in the study analysis unless the caregiver declines consent for that too.

For those neonates whose parents decline to consent to have their data included, study staff will assure the caregiver(s) that their neonates will continue to receive KNH standard care and will not be treated differently from all other neonates undergoing care in the unit. There will be no preferential treatment of study participants.

Informed consent to participate in onsite observations, IDI and/or FGD and to be audio-recorded will be obtained (Additional file 3). Confidentiality will be maintained during the discussions. Participants will also sign a non-disclosure clause within the informed consent form that prohibits discussions of the proceedings of the FGDs outside the group. Data from the discussions in audio recordings, transcripts and field notes will be treated confidentially and stored in laptops with passwords and in secured lockers at Aga Khan University, Nairobi. They will not be accessible to anyone outside the study team.

The results of this study will be shared with the Ministry of Health and submitted to local and international conferences and peer review journals.

## Discussion

There is a need to develop evidence-based and context-sensitive CPGs to standardise and improve the management of AOP in RCS. Concerns requiring resolution in implementing such CPGs include diagnosis of apnoea, optimal timing, dosing and administration of caffeine citrate, standardisation of monitoring devices and alarm limits and discharge protocols. We aim to provide a feasible standardised clinical care bundle for managing AOP in low- and middle-income settings.

A pragmatic approach reproducible in the RCS in real life is preferred because it does not utilise a controlled study setting. The mixed method formative study employing several qualitative data collection methods will allow a comprehensive understanding of the study context ‘hidden’ factors that may impede the uptake of clinical care bundle. The research team involved in this work comprises ‘insiders’ who have worked in the study hospital for many years (FM, GI). They have led the development and introduction of CPGs in RCS and conducted quality improvement research that contributed to the improvement of quality of care in the proposed study hospital. These experiences provide a comprehensive understanding of factors that influence the uptake of best practices [18, 28, 31, 32]. Further, the teaching faculty of the paediatric residents and neonatology fellows affiliated to the study hospital (FM, MW, GI) are perceived to be credible and thus well placed to motivate “buy-in” by the management and the frontline health workers in care improvement. Additionally, international collaborators in the research team (MA, ASG), will help change the mindset of the local experts in driving for ‘Zero Tolerance to Preventable Newborn Deaths’.

We envisage that PDSA and CQI strategies that employ audit and feedback, stakeholder engagement and application of the COM-B model will facilitate the uptake of best practices for the care of preterm infants beyond the quality indicators directly linked to our research goals. We are cognizant that this study may place more demand on human resources and essential commodities for the care of preterm infants beyond the scope of the research to sustain improved care outcomes. A deliberate effort will be made to engage the HCAs in the clinical audits to appreciate the need to budget for the extra demands. The flexibility and adaptability of the PDSA cycles and their innate nature of allowing new learning to build on the experimental process will support the adaptation of the AOP clinical-care-bundle prototype to make it appropriate for use in RCS.

## Supplementary Information

**Additional file 1.** Topic guides for IDIs and FGDs.

**Additional file 2.** Checklist of non-participant observation during the formative phase.

**Additional file 3.** Consent forms.

**Additional file 4.** REDCap tools.

**Additional file 5.** ERC.

**Additional file 6.** ERC renewal.

## Data Availability

De-identified data available on request.

## Abbreviations

AOP: Apnoea of prematurity
CPAP: Continuous positive airway pressure
CPGs: Clinical practice guidelines
COM-B: Capability, Opportunity, and Motivation model of behavior change
CQI: Continuous quality improvement
FGD: Focus group discussions
GA: Gestational age
HCA: Health care administrators
HCPs: Health care practitioners
IDI: In-depth interviews
NBU: Newborn unit
PDSA: Plan-Do-Study-Act
RA: Research assistant
RCS: Resource-constrained setting
REDCap: Research Electronic Data Capture
SpO_2_: Peripheral oxygen saturation
WHO: World Health Organization
