# Supplementary material for: Developing and testing a clinical care bundle incorporating caffeine citrate to manage apnoea of prematurity in a resource-constrained setting: a mixed methods clinical feasibility study protocol
Source: Implement Sci Commun. 2023 Jul 17;4:80. doi: 10.1186/s43058-023-00455-x (PMC10351121; doi:10.1186/s43058-023-00455-x)
Supplement: Supplementary file 1 — Additional file 1. Topic guides for IDIs and FGDs. [file 43058_2023_455_MOESM1_ESM.pdf]

**‘Evaluation of Technologies for Neonates in Africa (ETNA)**
**Caffeine for Apnea of Prematurity Feasibility Study**

**Caregivers Focus Group Discussion Guide**

Date of FGD______________________

Moderator _______________________

Notetaker________________________

Start time_________________________

End time__________________________

“Hello, my name is …………………. and my colleague’s name is (colleague to say name) and we are working on a project conducted here at the KNH that is examining the kind of care given to preterm babies in this unit. We would like to hear about your experiences and learn from your thoughts and feelings on the care given to the preterm babies. We will keep what you tell us today confidential, which means that nothing you say will be directly linked to you so please feel free to share. We do not work in this facility and your individual views will not be shared with others outside the study team. The responses you give to us will not in any way affect the care that you and your baby will receive in this unit, today or in future and will be used solely for purpose of improving care of new born babies. If you feel uncomfortable with any questions, let us know and we will skip it. You are also at liberty not to answer any questions you are uncomfortable with. Your views are very important as they will assist us improve care given to babies in this institution. My colleague will assist me in taking notes of our discussions and we wish to request that you allow us to audio-records the discussion so we do not miss out on any of your contributions. Before we start, do you have any questions for me? Is it ok to begin? Thank you, I will start the audio-recording now.”

1. As an icebreaker – each respondent to be asked to say their name, name of the baby, age of their baby, the birth order of their baby, if she has admitted to NBU KNH previously. The name they prefer to be called by during this discussion.

   (Note to moderator - here after refer the mothers and baby’s by the names they prefer.)

   **We now focus on your baby and your own experience.**
   **We appreciate each person may have different experiences or may be similar in some aspects – this statement may be repeated as necessary eg when there is silence or respondents struggling to discuss their experiences.**

2. Have you been made aware the reason for your baby’s admission into the NBU?

   *Probe:*
   *- Who informed you about the reasons for your baby’s admission? KNH NBU team? Maternity team? Referring hospital?*
   *When were you informed? On admission day or after?*
   *- What kind of information have you been provided with?*
   *- in your opinion was the information provided to you clear?*

3. Have you been informed about the care that your baby is getting and how long it will take?

*Probe:*

- *Have you been informed about the procedures that your baby will receive while in the NBU?*
- *What do you think about the care and the procedures provided to your baby? In your opinion do you think the care is adequate? Probe likes and dislikes*

4. Preterm babies by virtue of being born too soon require some special care compared to those baby’s born at term. For this discussion we shall focus on the special care given to preterms to enable them breath well or prevent them from developing breathing problems.

   *Are there problems related to breathing that a preterm baby might develop? – you might have known this previously or learned during this admission?*

   **(Moderator to check - what names do they give this condition – what do they call apnea- here after refer to apnea by the name agreed upon with the mothers)**

   *What kinds of information have you been provided with regarding (use term used by mothers for apnea) in pre-terms?*

   *Have you been informed about the medicines that your baby will receive to help preventing ‘apnea’?*

   *Probe: Do you have any concerns about this medication? What do you like about it? What don’t you like about it?*

   **Note to moderator: Next question is for mothers whose babies have developed apnea- actual intervention for apnea is ventilation – but our interest is the medication which in KNH is either caffeine or aminophylline)**

*Have you been informed about the medicines that your baby is receiving to help in treating and preventing more 'apneic attacks* **(Mod to use term identified by mothers)**
*Probe: Do you have any concerns about this medication? What do you like about it? What don't you like about it?*

*Have you been informed how your baby's progress will be monitored to identify any 'apneic episode'* **(Mod to use term identified by mothers)***?*

*Probe: Do you have any concerns about the way your baby will be monitored? What do you like about it? What don't you like about it?*

*For the period you have in in the NBU, what concerns (and worries) do you have about the care, 'to treat apnea'***(Mod to use term identified by mothers)** *that your baby is receiving?*

5. Would you have a problem if your baby, whose has not yet developed 'apnea' (**(Mod to use term identified by mothers)** is given medication to prevent 'apnea' immediately after birth? – by that we mean, that the doctor does not wait to see if your baby develops 'apnea' but gives all preterms who at very high risk of developing 'apnea'?

   *Probe:*
   - *Would you have any concerns about this preventative approach? If yes which concerns?*
   - *What new information would you wish to know about this medication before it is given to your baby?*

6. While in the NBU, sometimes very small babies at risk of breathing problems may be connected to a machine for close monitoring before and during certain treatments. This machine looks like this: **(mod to show machine to the group and explain/demonstrate briefly how it is connected to the baby)** Would you have any concerns or worries if your child is placed on such monitors?

*Probe*

*Would you have any concerns about your child being placed on such a machine? If yes which concerns?*
*What information would you like to have before or when your child is on the monitoring machine?*

7. Would you have any suggestions on how your experience during the care of your baby at this facility could be made more bearable/positive?

<u>Conclusion</u>

We have now come to the end of our discussion for today. Is there anything else you would like to talk about or a comment you wish to make?

Do you have any other questions for us?

Moderator: Respond to all questions asked, thank the participants and conclude the discussion.

**Evaluation of Technologies for Neonates in Africa (ETNA)**
**Caffeine for Apnea of Prematurity Feasibility Study**
**In-Depth Interview Guide – Healthcare Administrator (HCA)**

| **Administrative information** | |
|---|---|
| **HCA ID number:** | **Sex:** ☐ Female ☐ Male |
| **Date HCA informed consent form (ICF) signed:** \|D\|D\| - \|M\|M\|M\| - \|Y\|Y\|Y\|Y\| | |
| **HCA ICF signed prior to any study questions?** ☐ Yes ☐ No | |
| **Name of research staff who explained the ICF:** | |
| **HCA agreed to be audio-recorded?** ☐ Yes ☐ No<br>If No, why was the interview not recorded? ________________________________________________ | |
| **Name of interviewer:** | |
| **Date of interview:** \|D\|D\| - \|M\|M\| - \|Y\|Y\|Y\|Y\| | |
| **Interview start time:** \|H\|H\| : \|M\|M\| | |
| <u>**Instructions for qualitative research staff**</u>:<br>• Conduct the interview in the language with which the HCA feels most comfortable.<br>• Please **record the interview** using the audio-recorder (if HCA consent is provided) and state the ETNA HCA ID number. | |

| **Script to initiate the interview** |
|---|
| *"Hello, my name is __________ and I am a researcher with the ETNA project examining the management of "preterms" at KNH/your facility. We want to hear about your experiences and learn from your thoughts and feelings. We will keep what you tell us today confidential, which means that nothing you say will be directly linked to you so please feel free to share. If you feel uncomfortable with any question, let me know and we will skip it. Before we start, do you have any questions for me? Is it ok to begin? Thank you, I will start the discussion and turn on the audio-recorder'.* **(Skip last part for those who object to audio-recording)** |

| **A. Demographic information** |
|---|
| 1. First, we will start with some questions about yourself. What is your age? / How old are you?<br>2. What is your highest level of training completed? What is your background/designation? (e.g., hospital administrator, business administration, public health specialist, physician, etc.)<br>3. How long have you worked as a hospital administrator (anywhere)?<br><br>4. (*Ask Pharmacist only)* How long have you worked for (in) a newborn unit? (anywhere) |

| **B. Role and facility** |
|---|
| 1. How long have you been employed at or worked for KNH?<br>2. What is your current role and responsibilities at the KNH? *Probes: Please share what a typical day as an administrator is like for you.*<br>3. What challenges do you encounter in your duties at KNH?<br>4. We have observed that many babies in the newborn unit are pre-term. In this study we are mainly interested in discussing the care for the "preterms": **(only ask pharmacists this question)** What are some of the common health complications affecting "preterms" that you are familiar with?<br>5. In your routine work, what role do you play in supporting the management of preterm babies? |

| C. Management of apnea of prematurity |
|---|

For Pharmacists

*(Check if Apnea is mentioned at section by Q 4 above)* Among the complications of preterm babies is Apnea. Are you familiar with this condition? If yes, what does it mean to say a Pre-term has Apnea?

1. What drugs are routinely used for the management of Apnea?
2. Who is involved in determining the kinds of drugs to be used for apnea at KNH? What is used to determine the drug that is used for management of Apnea?
3. How are these drugs for the care of Apnea obtained? What is the process for the procurement of these drugs? Is this process the same for all other drugs?
4. How are the quantities of drugs to be procured determined? Who determines what quantities are procured?
5. How often are these drugs procured? Who determines how often the drugs are procured? What factors are considered when procuring these drugs?

*Probes: If aminophylline is mentioned, please probe further about barriers and facilitators to its use:*

- What are the barriers to the use of aminophylline in the management of apnea?

*Probes: if not mentioned probe barriers around:*

- *availability*
- *accessibility*
- *knowledge/ awareness*
- *human resources/ nursing staff, etc.*
- *costs*
- *Does the use of aminophylline pause any specific storage issues? If yes please explain*

6. Are there any guidelines in the procurement of drugs and other related equipment for use in the management of Apnea?

*Probes: If so, do you have a copy? Can I see? (If shown, please take a picture) if so, do you and your staff use them? If not, why not? If no policies or guidelines are in place, do you think there should be?*

**Procurement and tender committee officers**

In this study we are mainly interested in discussing the care for the “preterms” because we have observed that many babies in the newborn unit are pre-term.

1. How are drugs for the care of preterm babies procured? What is the process for the procurement of drugs for preterm babies? Is this process same for all other drugs?
2. Who determines the kinds of drugs to be procured for different conditions at the Newborn unit?
3. What drugs are routinely used for the management of Apnea?
4. What determines the drug that is procured for management of Apnea?

5. How are the quantities of drugs to be procured determined? Who determines what quantities are procured?
6. How often are these drugs procured? Who determines how often the drugs are procured? What factors are considered when procuring the drugs?

*Probes: If aminophylline is mentioned, please probe further about barriers and facilitators to its procurement:*

7. What are the barriers to the procurement of aminophylline in the management of apnea?

*Probes: if not mentioned probe barriers around:*

- *availability*
- *accessibility*
- *knowledge/ awareness*
- *human resources/ nursing staff, etc.*
- *costs*
- *In your view would aminophylline pose any storage problems once procured? If yes please explain*

10. Are there any guidelines in the procurement of drugs and other related equipment for use in the management of Apnea?

*Probes: If so, do you have a copy? Can I see? (If shown, please take a picture) if so, do you and your staff use them? If not, why not? If no policies or guidelines are in place, do you think there should be?*

**11. Caffeine citrate use in the newborn unit**

Section for Pharmacists

*(Check if Caffeine is mentioned in C above. If not inform: Caffeine is one of the drugs used for the management of Apnea. Are you familiar with its use? If answer is No, skip to Q 11)*

1. How is caffeine procured in the KNH and specifically to the NBU unit? How is the amount to be procured determined? Who is involved in this process?

*Probe: Are these procurement procedures similar to those of the other drugs used in the NBU? If not what is the difference?*

*2.* How frequently do you order caffeine for use at KNH (and for use at NBU?) What quantities do you normally order? What informs the quantities that you order? How often do you fail to get the orders you request for?

*Probe: what happens when you do not get the quantities of caffeine you ordered?*

*3.* Does the KNH experience stock-outs of caffeine citrate? If so, what happens when caffeine is out of stock? How often do the stockouts occur – check for any documentation of stock-outs? Are stock outs for other drugs common- are stock outs and their durations documented? Can I see any of that documentation? please ask for evidence?

4. Do you think it is feasible (check if the each of the feasible actions is sustainable) to rely on use of caffeine in the prevention and treatment of apnea of prematurity as standard treatment?

*Probe:*

- *Procurement*
- *availability*
- *storage,*
- *costs*
- *supply*

5. What are the barriers to the procurement of Caffeine in the management of apnea?

   *Probes: if not mentioned probe barriers around:*
   - *availability*
   - *accessibility*
   - *knowledge/ awareness*
   - *human resources/ nursing staff, etc.*
   - *costs*
   - In you view would caffeine pose any storage problems once procured? If yes please explain

6. What are your concerns/drawbacks in the procurement of caffeine, personally (if any?) What are your questions around caffeine procurement in the management of apnea of prematurity?

7. What do you think are potential unresolved issues around the use of caffeine in the prevention and treatment of apnea of prematurity at this hospital?

   *Probes:*
- *Availability of clinical guidelines /adherence to guidelines*
- *Availability of caffeine*
- *Ease of administration of caffeine*

8. What challenges/obstacles do you face when ordering caffeine? What would make it easier to regularly order caffeine for the required duration in babies in the management of apnea?

9. What would be required to get caffeine to be used routinely for all babies at risk of apnea and those with apnea at this facility.

   Probe: What would it take to make the use of Caffeine sustainable?

10. What would be required to get caffeine to be used routinely for all babies at risk of apnea and those with apnea at this facility.

    Probe: What would it take to make the use of Caffeine sustainable?

For <u>procurement and tender committee members ask the following</u>

*(Check if Caffeine is mentioned in C above. If not inform: Caffeine is one of the drugs used for the management of Apnea. Are you familiar with its use?) If answer is No, skip to Q 11.*

1. How is caffeine procured in the KNH and specifically to the NBU? How is the amount to be procured determined? Who is involved in this process?

*Probe: Are these procurement procedures similar to those of the other drugs used in the NBU? If not what is the difference?*

2. How frequently do you procure caffeine for use at KNH? What quantities do you normally order? What informs the quantities that you order?

3. In you view would caffeine pose any storage problems once procured?

4. What are your concerns/drawbacks in the procurement of caffeine, personally (if any?) What are your questions around caffeine procurement in the management of apnea of prematurity?

5. What do you think are potential unresolved issues around the procurement of caffeine in the prevention and treatment of apnea of prematurity at this hospital?

Probes:
- *availability of caffeine*

6. What challenges/obstacles do you face when procuring caffeine? What would make it easier to regularly procure caffeine for the required duration in babies in the management of apnea?

11. Taking into account the issues we discussed today, what would be your recommendation for caffeine use in the management of apnea of prematurity at this facility?

| **E. Closing** |
|---|
| Do you have any other comments about the management of apnea of prematurity or caffeine use or any other comments or concerns overall that we did not get to talk about and you would like to raise?<br><br>Do you have other questions regarding the interview we have had with you? If so, please ask them now<br><br>Note to interviewer: Please respond to all the questions asked before concluding the interview |
| *"Thank you for your time and the helpful information you have provided. Your feedback, along with feedback from other people we talk to will be used to recommend solutions for better care."* |
| **Interview end time:** \|H\|H\| : \|M\|M\| |

**Evaluation of Technologies for Neonates in Africa (ETNA)**
**Caffeine for Apnea of Prematurity Feasibility Study**
**In-Depth Interview Guide – Health Care Providers in NBU & Nursing Officer In-charge of NBU**

| **Administrative information** | |
|---|---|
| **ID number:** | **Sex:** ☐ Female ☐ Male |
| **Date respondent informed consent form (ICF) signed:** \|D\|D\| - \|M\|M\|M\| - \|Y\|Y\|Y\|Y\| | |
| **Respondent ICF signed prior to any study questions?** ☐ Yes ☐ No | |
| **Name of research staff who explained the ICF:** | |
| **Respondent agreed to be audio-recorded?** ☐ Yes ☐ No<br>If No, why was the interview not recorded? ______________________________ | |
| **Name of interviewer:** | |
| **Date of interview:** \|D\|D\| - \|M\|M\| - \|Y\|Y\|Y\|Y\| | |
| **Interview start time:** \|H\|H\| : \|M\|M\| | |
| <u>**Instructions for qualitative research staff**</u>:<br>• Conduct the interview in the language with which the **Respondent** feels most comfortable.<br>• Please **record the interview** using the audio-recorder (if **Respondent** consent to audio-record the interview is provided) and state the ETNA Respondent ID number. | |

**Script to initiate the interview**

*"Hello, my name is __________ and I am a researcher with the ETNA project examining the management of "preterms" at KNH/your facility. We want to hear about your experiences and learn from your thoughts and feelings. We will keep what you tell us today confidential, which means that nothing you say will be directly linked to you so please feel free to share. If you feel uncomfortable with any question, let me know and we will skip it. Before we start, do you have any questions for me? Is it ok to begin? Thank you, I will start the discussion and turn on the audio-recorder'.* **(Skip last part for those who object to audio-recording)**

**A. Demographic information**

1. First, we will start with some questions about yourself. What is your age? / How old are you?
2. What is your highest level of training completed? What is your background/designation? (e.g., Nurse, Neonatal nurse, medical officer, Paediatric Resident, Paediatrician, Neonatologists etc.)
3. How long have you worked in your current designation in NBU (anywhere)? How long have you worked in a newborn unit? (anywhere)

**B. Healthcare provider role and facility**

1. How long have you been employed at KNH?
2. How long have you worked in the KNH newborn unit?
3. What is your current role and responsibilities at the KNH newborn unit?
   *Probes: Please share what a typical day as an administrator is like for you. (to the NO in-charge- Are you also involved in patient care?) If yes, please explain your patient care responsibilities.*
4. We have observed that many babies in the newborn unit are pre-term. In this study we are mainly interested in discussing the care for the "preterms": What are some of the common health complications affecting "preterms" that you routinely encounter in this unit?
5. What makes care for "preterms" difficult? What would make care for "preterms" better in the unit?

| **C. Identification and management of apnea of prematurity** |
|---|
| 1. *(Check if Apnea is mentioned at 4)* Among the complications of preterm babies is Apnea. What does apnea of prematurity mean to you? What does it mean if you say a preterm has apnea? *(Please use the respondent's term in follow-up questions)*<br>2. Approximately how many "preterms" experiencing apnea of prematurity do you attend to ***(if the NBU in-charge ask how many "preterms" experiencing apnea of prematurity does this NBU typically see and take care of)*** in a week?<br>__________________________________<br>3. Which babies would you consider to be at risk of apnea of prematurity?<br>4. How do you identify preterms" **at risk** of apnea?<br>5. How do you manage "preterms" **at risk** of apnea of prematurity?<br><br>*Probes: Are any drugs used in the management? Which drugs and how often? If caffeine or aminophylline are mentioned, please probe further about why one drug is used vs the other. If caffeine or aminophylline are mentioned, please probe further about barriers and facilitators to their use:*<br><br>- *availability*<br>- *accessibility*<br>- *knowledge/ awareness*<br>- *human resources/ nursing staff, etc.*<br>- *Any specific monitoring activity to identify those who develop apnea of prematurity?*<br>______________________________________<br>6. How do you identify "preterms" **with** apnea of prematurity ?<br>7. How do you manage "preterms" **with** apnea of prematurity?<br><br>*Probes: Are any drugs used in the management? Which drugs and how often? If caffeine or aminophylline are mentioned, please probe further about why one drug is used vs the other. If caffeine or aminophylline are mentioned, please probe further about barriers and facilitators to their use:*<br><br>- *availability*<br>- *accessibility*<br>- *knowledge/ awareness*<br>- *human resources/ nursing staff, etc.*<br>- *Any specific monitoring activity to identify those who develop recurrent apnea of prematurity while on caffeine or aminophylline?*<br><br>________________________________________<br><br>8. Are you aware of any policies and/or guidelines for the management of apnea of prematurity?<br>*Probes: Are there guidelines for management of preterms at risk of apnea of prematurity? Are there guidelines for management of apneic episodes in preterms? If so, do you have a copy ( for both conditions)? Can I see them? (If shown, please take a picture) if so, do you and your staff use them? If not, why not? Do the guidelines specific the monitoring requirements for preterms on caffeine? If no policies or guidelines are in place, do you think there should be?* |
| |

| **D. Caffeine citrate use in the newborn unit** |
|---|
| 1-4 to be answered by the Nursing Officer In-charge of NBU, SKIP 1-4 when interviewing the HCP<br>1. How is caffeine used in this unit procured? How is the amount to be procured determined? Who is involved in this process?<br><br>*If nursing officer in charge is involved, probe: Are these procurement procedures similar to those of the other drugs used in the NBU? If not what is the difference?*<br>*2.* Does the KNH experience stock-outs of caffeine citrate? If so, what happens when caffeine is out of stock? How often do the stockouts occur – check for any documentation of stock-outs? Are stock outs for other drugs common- are stock outs and their durations documented? Can I see any of that documentation? please ask for evidence?<br><br>*3.* How frequently do you order caffeine from the pharmacy for this unit? What quantities do you normally order? What informs the quantities that you order? How often do you fail to get the orders you request for?<br><br>*Probe: How do you feel when you do not get the quantities of caffeine you ordered?*<br><br>4. Do you think it is feasible (check if the each of the feasible actions is sustainable) to rely on use caffeine in the prevention and treatment of apnea of prematurity as standard treatment?<br>*Probe:*<br>- Procurement<br>- availability<br>- storage,<br>- costs<br>- supply<br>- In you view would caffeine pose any storage problems once procured?<br><br>5. What are your concerns/drawbacks in the use of caffeine, personally (if any?) What are your concerns around caffeine use in the prevention and treatment of apnea of prematurity?<br><br>6. What do you think are potential unresolved issues around the use of caffeine in the prevention and treatment of apnea of prematurity at this hospital?<br><br>Probes:<br>- *Availability of clinical guidelines /adherence to guidelines*<br>- *Availability of caffeine*<br>- *Ease of administration of caffeine*<br>- *Use of devices for monitoring occurrence of apnea for those at risk as well as recurrence of apnea for those on caffeine or aminophylline*<br><br>*7.* In your view, what is the clinical benefit of using caffeine in the prevention and treatment of apnea of prematurity?<br>*Probes:*<br>*a. Do you think caffeine improves quality of care? Probe ( e.g. mental and lung development )*<br>*b. Do you think caffeine improves outcomes? Probe (e.g. length of admission in NBU, reduces risk of death)* |

8. Do you think use of monitoring devices improves detection of apnea of maturity? Why or why not?

9. Have you received any education or training on the use of caffeine, including monitoring of babies while on treatment? If so, what type of training? If not, how would you suggest this would be best offered? Which parameters in the monitoring devices would suggest occurrence of apnea in preterms at risk of prematurity?

10. Is the use of caffeine for apnea of prematurity possible/practical at this unit? If so, how so? If not, why not?
 *probes:*
 *If* barriers *and facilitators to caffeine use were not discussed above, please probe here about barriers and facilitators to caffeine use at this facility (e.g., accessibility, knowledge/ awareness, education/ training, human resources/ nursing staff, etc). Probe also if treatment of ALL those as risk is a priority or is it after they have developed apnea is treatment prioritized.*

*11.* What challenges/obstacles do you face when using caffeine in the unit? What would make it easier to regularly use caffeine for the required duration in babies a) at risk of apnea i.e. for prevention of apnea and b) also probe use in treatment of babies with apnea
 (*For doctors probe what would make it easier for them to adhere to protocols for apnea of prematurity and for the nurses probe what would make it easier to administer caffeine as prescribed and secondly to monitor patients as per best practices of management of apnea of prematurity).*

12. What would be required to get caffeine to be used routinely for all babies at risk of apnea and those with apnea at this facility.

 Probe: What would it take to make the use of Caffeine sustainable? this practice?

*Probes: Imagine if caffeine use was to used throughout for all babies who need it at this facility,*

- *What would be your reaction? Why?*
- *How do you think caregivers (mothers, parents, guardians, etc.) would react? Why?*
- *Integration into current flow of hospital operations, acceptance by administrators, etc.?*
- *What would be the specific issues that may arise with the switch or increased use of caffeine for the management of apnea of prematurity in particular given prophylactically for babies at risk?*
- *How would you suggest these issues be resolved?*
- *Let us focus specifically in service delivery in the NBU*
 *Are staff available to provide care that include best practices for management of apnea of prematurity?*
 *If a training package was to be developed for management of apnea of prematurity, which aspects of care for these babies should be emphasized in the training?*
 *What practical sessions should be included to build skills in the management of apnea of prematurity?*
 *What key information would you suggest being included in job aides, wall charts?*

13. Taking into account the issues we discussed today, do you have any recommendations for caffeine use in the prevention and treatment of apnea of prematurity at this facility?

| **E. Closing** |
|---|
| Do you have any other comments about prevention and treatment of apnea of prematurity or caffeine use or any other comments or concerns overall that we did not get to talk about and you would like to raise?<br><br>Do you have other questions regarding the interview we have had with you? If so, please ask them now<br><br>Note to interviewer: Please respond to all the questions asked before concluding the interview |
| *"Thank you for your time and the helpful information you have provided. Your feedback, along with feedback from other people we talk to will be used to recommend solutions for better care."* |
| **Interview end time:** \|H\|H\| : \|M\|M\| |
