# Supplementary material for: Developing and testing a clinical care bundle incorporating caffeine citrate to manage apnoea of prematurity in a resource-constrained setting: a mixed methods clinical feasibility study protocol
Source: Implement Sci Commun. 2023 Jul 17;4:80. doi: 10.1186/s43058-023-00455-x (PMC10351121; doi:10.1186/s43058-023-00455-x)
Supplement: Supplementary file 2 — Additional file 2. Checklist of non-participant observation during the formative phase. [file 43058_2023_455_MOESM2_ESM.pdf]

Checklist of non-participant observation during the formative phase

Name of Research assistant making observations____

**Activity**	**Observations**	**Remarks**
Gestational age determination<br>a) How is the gestational age determined? (e.g., calculated from birth weight, extracted from transfer from referral notes, etc.)?<br>b) If an ultrasound was done, when was it done?<br>c) Is a Ballard score done? If so, please observe. Is there documentation?		
Weight determination<br>a) At admission, is the infant weighed or are previously recorded weights used?<br>b) Who weighs the infant?<br>c) Where and when is weight recorded?<br>d) Is the weighing scale accessible when needed (e.g., at the point of admission)?<br>e) Is the weighing scale calibrated?<br>f) Is infant warmth maintained?<br>g) What is the timing of weighing in relation to feeding times?<br>h) Are infection, prevention and control (IPC) measure observed (e.g., handwashing before and after, disinfecting the scale before and after)?		
NGT insertion<br>a) Who prescribes nasal gastric tube (NGT) insertion?<br>b) Is family-centered care implemented prior to NGT insertion?<br>c) How long after the prescription is the NGT inserted?<br>d) Who inserts NGT?<br>e) Is the tube sized?<br>f) How is the position of the tube ascertained after insertion?<br>g) Is the NGT insertion documented?<br>h) Is infant warmth maintained?		

i) Are IPC measures observed (e.g., handwashing before and after)?		
NGT feeding<br>a) Is the position of the tube ascertained before a feed?<br>b) Who administers NGT feeds?<br>c) What are the timings for NGT feedings? Are the NGT feedings regular?<br>d) If irregular NGT feedings are observed, what are the causes?<br>e) Is infant warmth maintained?<br>f) Are IPC measures observed (e.g., handwashing before and after)?		
IV (intravenous) insertion<br>a) Is IV insertion prescribed as a procedure?<br>b) Is family-centered care implemented prior to IV insertion?<br>c) Who prescribes the IV?<br>d) Who inserts the IV, and how is it inserted (e.g., location on the body of insertion, use of vein finder, duration of insertion process, level of difficulty, etc.)?<br>e) How long after the IV prescription is the IV inserted?<br>f) Is the IV insertion documented?<br>g) Is infant warmth maintained?<br>h) Are IPC measures observed (e.g., cleaning the skin, letting skin dry, not touching the insertion site or needle etc.)?		
CPAP<br>a) Is CPAP given prophylactically for all preterms birthweight 1-3kg?<br>b) Who prescribes the rescue CPAP?<br>c) How long does it take for rescue CPAP to be initiated from the time it is prescribed?<br>d) Is rescue CPAP given on time for all who require it? i.e. not achieving SpO_2 (peripheral oxygen saturation) above		

90% while on oxygen 1l/min nasal prongs. No room for using Non-Rebreather Mask!<br>e) Who attaches the babies to CPAP?<br>f) Is the nasal prong sized?<br>g) How long does it take to prepare the baby for CPAP, prepare equipment and initiate CPAP?<br>h) Does the nasal prong fit snugly?<br>i) What is the current water level?<br>j) What is the current total flow rate?<br>k) What is the current fraction of inspired oxygen?<br>l) What is the current SpO_2? Do unexpected values raise a concern?<br>m) Is infant warmth maintained?<br>n) Are IPC measures observed (e.g., cleaning the skin, letting skin dry, not touching the insertion site, etc.)?<br>o) Practice of family-centered care?<br>p) What brand of CPAP is in use?		
Medication prescription and administration<br>a) Who prescribes medications?<br>b) Is the medication prescription adequately documented (e.g., including signature)?<br>c) Is there any reference to dosage charts or simple calculation without reference to guidelines?<br>d) Who administers IV/oral medication?<br>e) Are the prescriptions given on time (e.g., every 12 hours given +/-2hr, every 6 hourly given +/1 hour, stat doses given within 1hr)?<br>f) Is the medication administration adequately documented (e.g., including time)?<br>g) Is infant warmth maintained?<br>h) Are IPC measures observed (e.g., handwashing before and after medication procedure)?<br>i) Does the doctor or nurse explain the reason for medications and possible side effects?<br>j) Family Centered Care?		

Blood works<br>a) Who orders blood works?<br>b) Are blood works instructions clearly written (e.g., what test, when to give, etc.)?<br>c) How is the communication of this given from the decision-maker to the rest of the team?<br>d) How is the timeliness of blood work procedures compared with the time of order (e.g., point-of-care diagnostics within 30 min, others within 1hhour)?<br>e) Is the blood work process documented (e.g., if first done on rough paper and then transferred or documented from memory; time interval, etc.)?<br>f) Is infant warmth maintained?<br>g) Are IPC measures observed (e.g., handwashing before and after the procedure)?		
Monitoring<br>a) How are preterm infants <34 weeks gestational age monitored for apnea?<br>b) If intermittent monitoring, how often are preterms monitored?<br>• Respiratory Rate<br>• Heart Rate<br>• Oxygen saturation<br>c) Is infant warmth maintained during monitoring?<br>d) How is the documentation of monitoring done? When, where, by whom, and how is it done?<br>e) What is the duration between when observation is done, and the time it is documented? More than 1hour?		
Abnormal vital signs/alarms<br>a) Do abnormal vital signs or alarms cause concern to the nurses? If so, what do they do in response?<br>b) Is the doctor informed? If so, how soon and through what means was the doctor informed- face to face. Short Message Service, phone call?		

c) What is the duration between observing abnormal vital signs and doctor notification and doctors' response to notification?<br>d) Was the duration of apnea measured, and how and was it documented?		
Apnea identification<br>a) For infants <34 weeks gestational age, any method for monitoring the occurrence of apnea (e.g., proactive - routinely before apnea develops)?<br>b) How is the diagnosis of apnea made for infants who have been identified as having apnea? Are any causes attributed? Any documentation of the possible causes?<br>c) What actions are taken by nurses and by doctors when an infant develops apnea?<br>d) How is the duration of apnea measured?<br>e) Does the duration of apnea determine the nurse's response? The doctor's response?		
Apnea documentation<br>a) Are these apneic episodes documented? If so:<br>- Where are apneic episodes documented (e.g., Cardex, doctor continuation notes, observation charts, other)?<br>- Who documents apneic episodes?<br>- What is the interval between apnea occurrence and documentation? Is documentation completed immediately after the stabilization of the patient?<br>b) Are apnea interventions documented in the medical records, including time, intervention, and the infant status?		
Aminophylline or caffeine prescription and administration (**(State which of the two drugs observed):**		

a) Who prescribes aminophylline or Caffeine? Does the prescriber sign the prescription?<br>b) For dosage, any reference to drug guidelines or is the dose calculated?<br>c) Who administers the aminophylline or Caffeine?<br>d) Is the aminophylline or Caffeine administered as prescribed (e.g., 12 hourly given +/-2hour, 6 hourly given +/1 hour, stat doses given within 1 hour)?<br>e) Are any emergency life-saving drugs/ interventions given immediately, and by whom?		
Aminophylline or caffeine documentation **(State which of the two drugs observed):**<br>a) Is the aminophylline or caffeine prescription writing legible, and all details given as per the treatment chart (e.g., name of the drug, route of administration, dose, and frequency)? Is the duration of treatment prescribed and adhered to?<br>b) Is the aminophylline or caffeine administration documented, including when it is administered and by whom?<br>c) How is the documentation done? Is there any delay from the time it is administered to the time it is documented in the treatment chart?<br>d) If life-saving drugs are administered, are they documented within 1h of stabilizing the infant?<br>e) Does the doctor or nurse explain the reason for medications and possible side effects?		
Stopping Caffeine<br>a) When criteria were used to stop the drugs (prescribed duration over? Baby > 34 weeks, died, other)<br>b) Were instructions given on what to look out for after discontinuation of medications?<br>c) Was a follow-up appointment given and after how long in baby discharged		

Adverse events: **(State which of the two drugs observed):**<br>a) Were there any adverse events observed or recorded (e.g., tremors, opisthotonos, tonic-clonic seizures, vomiting, blood sugar > 7mmol/l, serum potassium < 3.5mmol/l or jaundice) that are attributed to the caffeine citrate?<br>b) Are the adverse events adequately documented (e.g., by whom, including time)?<br>c) Are the adverse events reported? To whom? Is the doctor notified? If so, are they reported and how long after the adverse event is noticed?<br>d) Does the adverse event raise any concern?<br>e) What actions are taken?		
Oxygen/CPAP/ventilatory support<br>a) Is the time documented when the decision is made to initiate oxygen, CPAP, and/or other ventilatory support and also when initiated?<br>b) Is the documentation adequate (e.g., condition of infant, action to be taken)?		
**Any other observations/comments:**		

Abbreviations: CPAP- Continuous airway positive pressure, IPC- infection prevention and control, IV- intravenous, NGT- Nasal gastric tube, SpO_2 - peripheral oxygen saturation
