# Supplementary material for: Developing and testing a clinical care bundle incorporating caffeine citrate to manage apnoea of prematurity in a resource-constrained setting: a mixed methods clinical feasibility study protocol
Source: Implement Sci Commun. 2023 Jul 17;4:80. doi: 10.1186/s43058-023-00455-x (PMC10351121; doi:10.1186/s43058-023-00455-x)
Supplement: Supplementary file 3 — Additional file 3. Consent forms. [file 43058_2023_455_MOESM3_ESM.pdf]

1.1 Informed Consent Forms

Caffeine Feasibility Study for Apnea of Prematurity at Kenyatta National Hospital

Caregiver and Neonate Participant Information and Informed Consent Form for Enrollment

Version 1.1, 09 February 2022

INVESTIGATORS

**Dr. Grace Irimu**	**Dr. Mary Waiyego**	**Dr. William Macharia**
Kenyatta National Hospital<br>Hospital Road<br>P O Box: 20723, Nairobi<br>Phone: +254-72-256-4600	Kenyatta National Hospital<br>Hospital Road<br>P O Box: 20723, Nairobi<br>Phone: +254-72-161-2393	Aga Khan University<br>3rd Parklands Avenue,<br>P O Box 30270, Nairobi<br>Phone: +254-20-366-1017

Part 1. Information Sheet

Introduction
You are being asked for you and your baby to take part in this study because your baby was born early and has been admitted to Kenyatta National Hospital. This study is sponsored by Aga Khan University and is funded by the Bill & Melinda Gates Foundation, an organization that promotes children's health and helps improve healthcare worldwide. The person in charge of this study at this hospital is Dr. Mary Waiyego.

This is a consent form that gives you information about the study and what will happen if you agree for you and your baby to be in the study. You are free to ask questions about the study at any time. If you agree to take part in this study, you will be asked to sign this consent form or make your mark/thumbprint in front of a witness. You will be given a copy of this form to keep. Another copy will stay with the study records.

Your and your baby's participation are completely voluntary. You have the right to decline to participate in this study or withdraw from this study at any time without negative consequences to you or your baby. You and your baby will receive the same care whether you participate in the study or not. Before you decide, you can talk to anyone you feel comfortable with about the research. If there is anything that you do not understand or you are concerned about the study, please ask the study staff at any time.

Why is this study being done?
In Kenya, there is a need to improve the health of babies born too early (premature). The goal of this study is to find the best way to provide safe care for babies like yours which can lead to improved clinical outcomes.

It is also important for us to understand what the parent/ caregiver thinks about the care options offered to your baby in a hospital setting. We may ask to interview you and/or for you to join a group discussion with other parents. By speaking with parents like you, we aim to learn more about the parent/ caregiver experience with and opinions about the care you and your baby have received.

What do we expect to learn from this study?
From this study we expect to learn how to take better care of small babies who stop breathing at this hospital.

What do I have to do if I take part in this study?
If you agree for you and your baby to be in the study, your baby will continue to receive the usual care the hospital provides. In addition, study nurses will observe and collect data from your baby's medical records. We may also invite you to discuss your experience with and opinions about the care you and your baby have received. However, no study activities or data collection will occur until you agree to participate in the study. Only after you have read (or have read to you), discuss, and sign or make a mark/thumbprint on this form, will you and your baby be enrolled in this study.

Upon enrollment, a monitor may be placed on your baby to collect information on your baby's vital signs. The monitors are safe, already used in within the newborn unit for routine care, and do not pose any significant risk or harm to your baby. During your baby's time in the study, your baby will continue to receive the care recommended by the doctors without interruption.

At enrollment, you will be asked to do the following things if you decide you want your baby to be in the study:

- Answer questions about you, your baby, and your family.
- Tell the study staff about any medical problems you and your baby have had.
- Allow your baby's vital signs to be monitored.
- Allow documentation of clinical activities that take place while your baby is in the study (like medication or other treatments).
- Allow the study team to collect information about your baby from other hospital information sources.

In addition, during the study we may approach you and ask to interview you and/or for you to join a group discussion with other parents that will take about 45 to 90 minutes. If you agree to take part in an interview and/or group discussion, you will sit with a study staff member or in a group and discuss your experience participating in the research study. The study staff member might make an audio-recording of the interview. Again, no study activities will begin before they have been fully explained to you, you have let us know that you understand the study, and you have agreed to participate.

What are the alternatives to study participation?
You have the option to not participate in this study. There will be no negative consequences to you or your baby if you choose not to participate in this study. Should you choose not to participate, your baby will continue to receive the usual standard of care from the hospital staff.

You may choose to have your baby participate in the study but decline to participate in an interview or group discussion. You do not have to participate in an interview or group discussion to have your baby continue in the study.

Why would the study staff withdraw my baby from this study early?
The study doctor may need to take your baby out of the study early if:

- The study is stopped by the sponsor, funder, ethics committee, study protocol team or any other regulatory body.
- Your baby is discharged from the hospital or transfered.

- There are other reasons that may prevent you or your baby from completing the study successfully.

What are the risks of being in this study?
Answering some questions may make you feel uncomfortable. You are free to skip any questions that you do not want to answer. Some of the monitors used in the study may cause minimal discomfort to your baby but the monitors are safe, already used in Kenya, and do not cause significant risk or harm to your baby. Study staff will make sure to minimize any potential distress, and babies may not be enrolled in the study if study staff believes that including them in the study could delay or interrupt their care in any way.

Are there benefits to taking part in this study?
You and your baby will not receive any additional benefit from being in this study. This study is designed to help better understand how to provide safe and efficient care for premature newborn babies.

What about confidentiality?
All possible measures will be taken to keep your and your baby's personal information confidential. If this study is published, your and your baby's names will not be used, and you and your baby will not be personally identified. Any photographs or videos of your baby will not include your baby's face or identification.

In order to make sure this study is being done properly, your records may be reviewed by:
- Study staff and monitors
- Ethics committees and/or instituitional review boards

You and your baby's study records will be kept at the hospital for at least five years after the study is completed or for the duration required by Kenyan law. If you want the results of the study, let the study staff know that you would like them. If you decide to leave the study, information already collected from your baby will still be used for the study.

What are the costs to me?
There is no cost to you for your and your baby's participation in this study.

Will I receive payment?
You will not receive any payment for your and your baby's participation in this study.

What happens if my baby is injured during the study?
It is unlikely that you or your baby will be injured as a result of being in this study. If you have any concerns related to this study, please immediately contact Dr. Mary Waiyego, our study neonatologist, at 072-161-2393 and she will tell you where your baby can receive treatment.

What are my and my baby's rights as study participants?
Taking part or having your baby take part in this study is completely up to you. You may choose to withdraw you or your baby from study participation at any time. There will be no penalty or loss of benefits to which you and your baby are otherwise entitled. You and your baby will be treated the same no matter what you decide. If you choose to not have you or your baby be in the study, you and your baby will not lose the benefit of services to which you would normally have at this hospital.

We will tell you about new information from this or other studies that may affect you and your baby's health, welfare, or willingness to stay in this study. If you want the results of the study, let the study staff know that you would like them.

A description of this clinical trial will be available on http://www.ClinicalTrials.gov, as required by United States of America (USA) law. This website will not include information that could identify you and your baby. You can search this website at any time.

The research study was reviewed and approved by the Aga Khan University Research Ethics Committee, the Kenyatta National Hospital-University of Nairobi Ethics Research Committee, and the National Council for Science and Technology-National Bioethics Committee.

What do I do if I have problems or questions?
For questions, concerns, or complaints about the study, you or your baby's rights as study participants, or if your baby has a research-related injury, you should contact:

- Dr. Mary Waiyego, Kenyatta National Hospital, Hospital Rd, Nairobi. Tel: 072-161-2393; email: waiyegomary99@gmail.com
- Secretary/Chairperson, Kenyatta National Hospital-University of Nairobi Ethics and Research Committee Telephone No. 2726300 Ext. 44102 email uonknh_erc@uonbi.ac.ke.

Caregiver and Neonate Participant Information and Informed Consent Form for Enrollment

Part 2. Statement of Non-disclosure and Consent

Participant's statement

I have read this consent form or had the information read to me. I have had the chance to discuss this research study with the study staff. I have had my questions answered in a language that I understand. The risks and benefits have been explained to me. I understand that my participation in this study is voluntary and that I may choose to withdraw any time. I freely agree to participate in this research study. I understand that all efforts will be made to keep information regarding my personal identity confidential. By signing this consent form, I have not given up any of the legal rights that I have as a participant in a research study.

In addition, I agree to maintain the confidentiality of other study participant information disclosed during any group discussions or anything observed during my participation in study activities, and to hold in confidence any and all study participant proceedings observed directly or indirectly.

I agree to participate in this research study **Yes** **No**

Participant printed name

____

Participant signature / Thumb stamp

____

Date ___________________

Researcher's statement

I, the undersigned, have fully explained the relevant details of this research study to the participant named above and believe that the participant has understood and has willingly and freely given their consent.

Researcher's printed name ___________________________ **Date** _________________

Signature ______________________________________

Role in the study ___________________________

Witness Printed Name (If witness is necessary, a witness is a person mutually acceptable to both the study staff and the participant)

Name ________________________________ Contact information ____________________

Signature /Thumb stamp _______________________________ Date __________________

Caffeine Feasibility Study for Apnea of Prematurity at Kenyatta National Hospital

Maelezo ya mshiriki kwa mlezi na mtoto mchanga na fomu ya idhini/makubaliano kwa usajili

Toleo 1.1, 09 Februari 2022

WATAFITI

**Dr. Grace Irimu**	**Dr. Mary Waiyego**	**Dr. William Macharia**
Kenyatta National Hospital<br>Hospital Road<br>P O Box: 20723, Nairobi<br>Phone: +254-72-256-4600	Kenyatta National Hospital<br>Hospital Road<br>P O Box: 20723, Nairobi<br>Phone: +254-72-161-2393	Aga Khan University<br>3rd Parklands Avenue,<br>P O Box 30270, Nairobi<br>Phone: +254-20-366-1017

Sehemu ya 1. Karatasi ya maelezo

Utangulizi
Tunakuomba wewe na mtoto wako mushiriki kwenye utafiti huu kwa sababu mtoto wako alizaliwa mapema na amelazwa hapa hospitali ya Kitaifa ya Kenyatta. Utafiti huu unafadhiliwa ni Chuo Kikuu cha Aga Khan na Wakfu wa Bill & Melinda Gates, shirika ambalo linakuza afya za watoto na kusaidia katika kuboresha huduma za afya duniani kote. Mtu anayesimamia utafiti huu katika hospitali hii ni Dakrari Mary Waiyego.

Hii ni fomu ya idhini ambayo inakupatia maelezo kuhusu utafiti huu na kile ambacho kitafanyika kama utakubali wewe na mtoto wako kujiunga na utafiti huu. Uko huru kuuliza maswali kuhusu utafiti huu wakati wowote. Ukikubali kushiriki kwenye utafiti huu, utaombwa uweke sahihi yako kwenye fomu hii ya idhini/makubaliano ama uweke alama ya kidole gumba kwenye fomu mbele ya shahidi. Utapewa kopi moja ya fomu hii uweke. Kopi nyengine itabaki kwenye kumbukumbu za utafiti.

Kushiriki kwako na mtoto wako kwenye utafiti huu ni hiari kabisa. Uko na uhuru wa kukataa kushiriki au kujitoa kwenye utafiti huu wakati wowote bila ya wewe au mtoto wako kupata matokeo yoyote mabaya. Wewe na mtoto wako mutapata huduma sawa bila kujali iwapo mutashiriki kwenye utafiti huu au la. Kama kuna kitu chochote ambacho hukielewi kuhusu utafiti huu, tafadhali uliza wafanyikazi wa utafiti huu wakati wowote.

Ni kwa nini utafiti huu unafanywa?
Hapa inchini Kenya ipo haja ya kuboresha afya ya watoto waliozaliwa mapema (watoto waliozaliwa kabla ya wakati wao kufika). Lengo la utafiti huu ni kutafuta njia bora zaidi za kutoa huduma salama kwa watoto wachanga kama huyu wako ambazo zinaweza kuleta matokeo bora ya matibabu.
Pia ni muhimu kwetu kuelewa ni kitu gani ambacho mzazi/mlezi anafikiria kuhusu chaguzi/aina za utunzaji zinazotolewa kwa mtoto wako katika mipangilio ya hospitali. Tunaweza kukuomba tufanye mahojiano na wewe/au ushiriki kwenye majadiliano ya kikundi na wazazi wengine. Lengo la kuongea na wazaii kama wewe ni kujua zaidi kuhusu uzoefu wa mzazi/mlezi na mawazo yao kuhusu huduma ambayo wewe na mtoto wako mumepata.
Ni kitu gani ambacho tunatarajia kujifunza kutokana na huu utafiti?

Kutokana na utafiti huu tunatarajia kujifunza kuhusu jinsi ya kukuza njia salama na bora ili kutoa huduma zenye ubora wa hali ya juu kwa watoto wanaoacha kupumua katika hospitali hii.

Ni kitu gani nitafanya nikishiriki kwenye utafiti huu?
Ukikubali wewe na mtoto wako mushiriki kwenye utafiti huu, mtoto wako ataendelea kuhudumia kama kawaida. Kwa ziada, wafanyikazi wa utafiti huu wataangalia mtoto wako na recodi zake. Harakati za utafiti huu zitajumuisha kuangalia hali ya mtoto wako na kuuliza maswali kuhusu wewe na mtoto wako. Hakuna harakati za utafiti zitakazofanyiwa mtoto wako kabla hazijaelezwa kwako kikamilifu, utatuambia kama unazielewa harakati za utafiti huu, na umeweka sahihi ama kuweka alama ya kidole gumba katika fomu hii ya makubaliano/idhini. Wewe na mtoto wako mutasajiliwa kwenye utafiti huu tu baada ya kusoma (ama kusomewa), kujadili na kuweka sahihi au alama ya kidole gumba kwenye fomu hii.

Baada ya kusajiliwa kifaa cha kufuatilia kinaweza kuwekwa kwa mtoto wako ili kinakili maelezo kuhusu ishara muhimu za mtoto wako. Vifaa vya kufuatilia viko salalma, tayari vinatumika hapa hospitalini, na haviwezi kusababisha hatari kubwa au madhara kwa mtoto wako. Vifaa vya kufuatilia vinaweza kubaki kwa mtoto wako kwa muda wote ambao mtoto wako atakuwa hospitalini lakini havitaleta usumbufu wowote kwa matibabu ya kawaida kwa mtoto wako. Mtoto wako ataendelea kupata huduma iliyopendekezwa kutoka kwa wahudumu wa hospitali bila usumbufu wowote kwa muda wote ambao atakuwa kwenye utafiti huu.

Wakati wa kusajiliwa utaombwa kufanya mambo yafuatayo ukiamua kwamba mtoto wako ajiunge na utafiti huu:

- Ujibu maswali kuhusu wewe mwenyewe, mtoto wako na familia yako.
- Uwaambie wafanyikazi kwenye utafiti huu kuhusu matatizo yote ya kiafya ambayo wewe na mtoto wako mumekuwa nayo.
- Kuruhusu ishara muhimu za mtoto wako kufuatiliwa.
- Kuruhusu kunakiliwa kwa shughuli zote za matibabu ambazo zitafanyika wakati mtoto wako yuko kwenye utafiti huu (kama vile madawa au matibabu mengine)
- Kuruhusu wafanyikazi kwenye utafiti huu kuchukua maelezo kuhusu mtoto wako kutoka kwenye vyanzo vingine vya maelezo vya hospitali.

Kwa kuongezea, wakati utafiti unapoendelea unaweza kuombwa ufanyiwe mahojiano/ama ushiriki kwenye mahojiano ya kikundi na wazazi wengine ambayo yatachukua kati ya dakika 45 hadi 90. Ukikubali kushiriki kwenye mahojiano/ama kwenye majadiliano ya kikundi, utakaa na mfanyikazi wa utafiti huu/ama utakaa kwenye kikundi ili mujadili kuhusu munayoyapitia/muliyoyapitia kwenye utafiti huu. Mfanyi kazi kwenye utafiti huu anaweza kurekodi sauti zenu wakati wa majadiliano. Pia, hakuna shughuli za utafiti zitakazoanza kabla hazijafafanuliwa kwako kikamilifu, utatuambia iwapo umeuelewa utafiti huu na umekubali kushiriki.

Ni njia gani mbadala za kushiriki kkwenye utafiti huu?
Uko na chaguo la kutoshiriki kwenye utafiti huu. Hakutakuwa na matukio yoyote kwako wewe au kwa mtoto wako ukichagua kutoshiriki kwenye utafiti huu. Ukichagua kutoshiriki kwenye utafiti huu, mtoto wako ataendelea kupata matibabu ya kiwango cha kawaida kutoka kwa wafanyikazi wa hospitali.

Unaweza kuchagua kwamba mtoto wako ashiriki kwenye utafiti huu lakini ukatae kushiriki kwenye mahojiano ama majadiliano ya kikundi. Sio lazima ushiriki kwenye mahojiano ama kwenye majadiliano ya kikundi ndio mtoto wako ashiriki kwenye utafiti huu

Ni kwa nini wafanyikazi wa utafiti wanaweza kumtoa mtoto wangu kwenye utafiti mapema?
Daktari wa utafiti huenda akhitajika kumtoa mtoto wako kwenye utafiti huu mapema iwapo/kama:

- Kama utafiti utasimamishwa ni mfadhili, mwanzilishi, kamati ya kuangalia maadili mema ya utafiti ama chombo chengine cha udhibiti.
- Kama mtoto wako atatolewa hospitalini'
- Kama kutakuwa na sababu zingine ambazo huenda zitakuzuia wewe au mtoto wako kukamilisha utafiti huu kwa mafanikio

Kuna athari gani zinazotokana na kushiriki kwenye utafiti huu?
Kwa kuyajibu maswali mengine huenda ukahisi/ukajisikia una wasiwasi ama kuwa na usumbufu. Uko huru kuruka maswali yoyote ambayo hutaki kuyajibu. Baadhi ya vifaa vya kufuatilia vitakavyotumika huenda vikasababisha usumbufu kidogo kwa mtoto wako lakini vifaa hivi vya kufuatilia viko salama, tayari vinatumika hapa Kenya na haviwezi kusababisha hatari yoyote kubwa au madhara kwa mtoto wako. Wafanyikazi kwenye utafiti huu watahakikisha wamepunguza usumbufu wowote unaowezekana, na watoto wanaweza kukosa kusajiliwa kwenye utafiti iwapo wafanyikazi wa utafiti wataamini kwamba kuwajumuisha kwenye utafiti kunaweza kucheleweshа au kukatiza matibabu yao kwa njia yoyote.

Je, kuna manufaa yoyote ya kushiriki kwenye utafiti huu?
Wewe na mtoto wako hamtapata manufaa yoyote ya ziada kwa kushiriki kwenye utafiti huu. Utafiti huu umeundwa ili kusaidia kuelewa zaidi jinsi ya kutoa zilizo salama na zenye ufanisi kwa watoto waliozaliwa mapema kabla ya wakati wao kufika.

Je, ni vipi kuhusu usiri?
Hatua zote zinazowezekana zitachukuliwa ili kuweka siri maelezo ya kibinafsi yako na ya mtoto wako. Ikiwa utafiti huu utachapishwa, jina lako na la mtoto wako hayatatumika, na wewe pia mtoto wako binafsi hamtatambuliwa.

Ili kuhakikisha kwamba utafiti huu unafanyika vizuri, rekodi zako huenda zikakaguliwa ni:

- Wafanyikazi wa utafiti na wachunguzi,
- Kamati ya kuangalia maadili mema ya utafiti/ama bodi za ukaguzi wa kitaasisi

Rekodi zako na mtoto wako za utafiti zitawekwa hapa hospitalini kwa muda usiopungua miaka mitano baada ya utafiti kukamilika ama kwa muda unaohitajika kisheria ya Kenya. Kama utahitaji majibu ya utafiti huu, mjulishe mfanyikazi wa utafiti. Ukiamua kujitoa kwenye utafiti huu, maelezo ambayo yatakuwa yamepatikana kutoka kwa mtoto wako bado yatatumika kwenye utafiti huu.

Je, kuna gharama gani kwangu?
Hakuna gharama zozote kwako au kwa mtoto wako kwa kushiriki kwenye utafiti huu.

Je, nitapokea malipo yoyote?
Hautapokea malipo yoyote kwa wewe au mtoto wako kushiriki kwenye utafiti huu.

Ni nini kitakachotokea ikiwa mtoto wangu atajeruhiwa wakati wa utafiti?
Haiwezekani kwamba wewe au mtoto wako mutapata majeraha kwa kushiriki kwenye utafiti huu. Ukipata wasiwasi kwa sababu ya kushiriki kwenye utafiti huu, tafadhali wasiliana kwa

haraka sana na Daktari Mary Waiyego kupitia numbari ya simu 072-1612393 na atakwambia ni wapi ambako mtoto wako anaweza kupata huduma.

Je, haki zangu na za mtoto wangu ni zipi kama mshiriki wa utafiti?
Wewe ama mtoto wako kushiriki kwenye utafiti huu ni uamuzi wako wewe mwenyewe. Unaweza kuchagua mwanao kuacha kuendelea na taratibu za utafiti hii wakati wowote. – hautapata adhabu yoyote ama kukosa faida zozote ambazo wewe na mtoto wako munastahili kupata. Wewe na mtoto wako mutachukuliwa tu kama kawaida bila kujali uamuzi wako. Ukichagua wewe na mtoto wako kutoshiriki kwenye utafiti, wewe na mtoto wako hamtapoteza faida za kupata huduma ambazo mtapata kama kwaida katika hospitali hii.

Tutakuambia kuhusu habari mpya kutoka kwa utafiti huu au tafiti zingine ambazo huenda zikaaathiri afya yako na ile ya mtoto wako, ustawi, nia ya kutaka kubaki kwenye utafiti huu. Kama unataka majibu ya utafiti huu, mjulishe mfanyikazi kwenye utafiti huu

Maelezo kuhusu utafiti huu yatapatikana kwenye http://www.ClinicalTrials.gov, kama inavyohitajiwa kwa sharia ya United States of America (USA)
Utafiti huu ulipitiwa na kuthibitishwa ni Kamati ya Maadili Bora ya Utafiti ya Chuo Kikuu cha Aga Khan, na Baraza la Kitaifa la Sayansi na Teknologia- National Bioethics Committee.

Je nitafanya nini nikiwa na matatizo au maswali?

Ukiwa na maswali, wasiwasi, au malalamiko kuhusu utafiti huu, haki zako wewe au mtoto wako kama mshiriki wa utafiti, ama ikiwa mtoto wako ako na jeraha ambalo linahusiana na utafiti huu, unatakiwa kuwasiliana na:

- Daktari Mary Waiyego, Hospitali ya Kitaifa ya Kenyatta, Hospital Road Nairobi. Nambari ya simu 072-161-2393; barua pepe:waiyegomari99@gmail.com
- Mwandishi/mwenyekiti, Kamati ya Maadili ya Utafiti ya Hospitali ya Kitaifa ya Kenyatta-Chuo Kikuu cha Nairobi, nambari ya simu 2726300 Ext. 44102 email: uonknh erc@uoni.ac.ke.

Maelezo ya mshiriki kwa mlezi na mtoto mchanga na fomu ya idhini/makubaliano kwa usajili

Sehemu ya 2. Taarifa ya kutofichua na idhini/makubaliano

Taarifa ya washiriki

Nimesoma au nimesomewa fomu ya idhini/makubaliano. Nimepata nafasi ya kujadili utafiti huu na wafanyikazi wa utafiti. Maswali yangu yamejibiwa katika lugha ambayo naielewa. Nimeelezewa athari na faida za utafiti huu. Ninaelewa kwamba kushiriki kwangu katika utafiti huu ni kwa hiari yangu na pia ninaweza kuchagua kujitoa kwenye utafiti wakati wowote. Nimekubali kwa hiari yangu kushiriki kwenye utafiti huu. Ninaelewa kwamba kila mbinu itafanywa ili kuweka maelezo yote yanayonitambulisha kuwa siri. Kwa kusaini fomu hii ya idhini/makubaliano, sijaacha haki yoyote ya kisheria ambazo niko nazo kama mshiriki wa utafiti.

Kwa kuongezea, niakubali kudumisha usiri wa maelezo yaliyotolewa ni washiriki wengine kwenye utafiti wakati wa majadiliano ya kikundi ama kitu chochote nilichoona wakati nikishiriki kwenye shughuli za utafiti, na kushikilia kwa kujiamini na shughuli zote za utafiti nilizoona moja kwa moja au kwa njia isiyo ya moja kwa moja.

Nina kubali kushiriki kwenye utafiti huu **Ndiyo** **La**

Jina la mshiriki ---

Sahihi ya mshiriki/alama ya kidole gumba -----------------------------------

Tarehe-----------------------------------

Taarifa ya mtafiti

Mimi, niliyetia sahihi, nimeeleza kwa kikamilifu mambo yote yanayohitajika kwa utafiti huu kwa mshiriki aliyetajwa hapo juu na ninaamini kwamba mshiriki ameelewa na ametoa idhini/amekubali kwa hiari yake bila kushurutishwa/kushawishiwa

Jina la mtafiti--- Tarehe--------------------------

Sahihi ---

Jukumu lake kwenye utafiti--

Jina la shahidi (kama shahidi anahitajika, shahidi ni mtu ambaye anakubalika katika pande zote kwa mshiriki na utafiti)

Name ______________________________ Contact information ____________________

Jina ---   Maelezo ya mawasiliano -------------------------------------

Signature /Thumb stamp ______________________________   Date __________________

Sahihi/alama ya kidole gumba -----------------------------   Tarehe -------------------------

Caffeine Feasibility Study for Apnea of Prematurity at Kenyatta National Hospital

Healthcare Provider/ Administrator Participant Information and Informed Consent Form for Enrollment

Version 1.1, 09 February 2022

INVESTIGATORS

**Dr. Grace Irimu**	**Dr. Mary Waiyego**	**Dr. William Macharia**
Kenyatta National Hospital<br>Hospital Road<br>P O Box: 20723, Nairobi<br>Phone: +254-72-256-4600	Kenyatta National Hospital<br>Hospital Road<br>P O Box: 20723, Nairobi<br>Phone: +254-72-161-2393	Aga Khan University<br>3rd Parklands Avenue,<br>P O Box 30270, Nairobi<br>Phone: +254-20-366-1017

Part 1. Information Sheet

Introduction

You are being asked to take part in this study because you are a healthcare provider or administrator involved in the "Feasibility of management of apnea of prematurity with caffeine at a tertiary health care facility in Kenya. A quality improvement study." This study is sponsored by Aga Khan University and is funded by the Bill & Melinda Gates Foundation, an organization that promotes children's health and helps improve healthcare worldwide. The person in charge of this study at this hospital is Dr. Mary Waiyego.

This is a consent form that gives you information about the study and what you will have to do if you agree to be in the study. You are free to ask questions about the study at any time. If you agree to take part in this study, you will be asked to sign this consent form. You will be given a copy of this form to keep. Another copy will stay with the study records.

Your participation is completely voluntary. You have the right to refuse to join or withdraw from the study at any time without negative consequences to you or your employment. Before you decide to participate, you can talk to anyone you feel comfortable with about the research. If there is anything that you do not understand about the study, please ask the study staff or Dr. Waiyego at any time.

Why is this study being done?

In Kenya, there is a need to improve the health and survival of premature babies. A serious health concern, apnea of prematurity is when premature babies experience difficulty breathing, including stopping breathing for short periods of time. The goal of this study is to find the best way to provide safe, effective, and high-quality care which will lead to improved clinical outcomes for newborn babies with apnea of prematurity. As part of this study, we aim to learn more about healthcare providers' and administrators' experiences with and perceptions around introducing caffeine citrate as part of a clinical care bundle to manage apnea of prematurity. Healthcare providers and administrators involved in supporting premature neonates at this hospital during the study period may be asked to take part in this study.

What do we expect to learn from this study?

From this study, we expect to learn from healthcare providers and administrators their thoughts, attitudes, and practices around the introduction of caffeine citrate as part of a clinical care bundle to manage apnea of prematurity in this hospital as well as barriers and facilitators.

What do I have to do if I take part in this study?
If you agree to take part in the study, you may participate in one or more study discussions. A study visit may consist of an in-depth interview, participation in a focus group discussion, and/or direct observations. These study visit may last up to 90 minutes. No study activities will be initiated before they have been fully explained to you, you have let us know that you understand the study activities, and you have signed this consent form. Only after you have read, discussed, and signed this form, will you be enrolled in this study. After signing this consent form, you will be enrolled and you will participate in discussions or answer questions about your experience with and perceptions around providing caffeine for apnea of prematurity and the use of monitors. Audio-recordings may be made of the study visits.

What are the alternatives to study participation?
You have the option to not participate in this study. There will be no consequences to you or your employment if you choose not to participate in this study.

What are the risks of being in this study?
Answering some questions may make you feel uncomfortable. You are free to skip any questions that you do not want to answer.

Are there benefits to taking part in this study?
There are no direct benefits to you for taking part in this study. This study is designed to help better understand how to provide safe, efficient, and high-quality care for premature newborn babies in this hospital. This may benefit the clinical processes for hospital staff and clinical outcomes for babies in the future.

What about confidentiality?
All possible measures will be taken to keep your personal information confidential. All personal information during the study will be kept confidential. If this study is published, your name will not be used, and you will not be personally identified. Any photographs or videos will not include your face or identification.

In order to make sure this study is being done properly, your records may be reviewed by:
- Study staff and monitors
- Ethics committees and/or institutional review boards

Your study records will be kept at the hospital for at least five years after the study is completed or for the duration required by Kenyan law. If you want the results of the study, let the study staff know that you would like them. If you decide to leave the study, information already collected from you will still be used for the study.

What are the costs to me?
There is no cost to you for your participation in this study.

Will I receive payment?
You will not receive any payment for your participation in this qualitative study.

What are my rights as a study participant?

Taking part in this study is completely voluntary and you may choose to withdraw your participation at any time. If you choose not to be in the study, there will be no penalty to you or to your employment. We will tell you about new information from this or other similar studies that may affect your willingness to stay in this study. If you want the results of the study, let the study staff know that you would like them.

A description of this clinical trial will be available on http://www.ClinicalTrials.gov, as required by United States of America (USA) law. This website will not include information that could identify you. You can search this website at any time.

The research study was reviewed and approved by the Aga Khan University Research Ethics Committee, the Kenyatta National Hospital-University of Nairobi Ethics Research Committee, and the National Council for Science and Technology-National Bioethics Committee.

What do I do if I have problems or questions?
For questions, concerns, complaints about the study or your rights as a study participant, you should contact:

- Dr. Mary Waiyego, Kenyatta National Hospital, Hospital Rd, Nairobi. Tel: 072-161-2393; email: waiyegomary99@gmail.com
- Secretary/Chairperson, Kenyatta National Hospital-University of Nairobi Ethics and Research Committee Telephone No. 2726300 Ext. 44102 email uonknh_erc@uonbi.ac.ke.

Caffeine Feasibility Study for Apnea of Prematurity at Kenyatta National Hospital

Healthcare Provider/ Administrator Participant Information and Informed Consent Form for Enrollment

Part 2. Statement of Non-disclosure and Consent

Participant's statement

I have read this consent form or had the information read to me. I have had the chance to discuss this research study with the study staff. I have had my questions answered in a language that I understand. The risks and benefits have been explained to me. I understand that my participation in this study is voluntary and that I may choose to withdraw any time. I freely agree to participate in this research study. I understand that all efforts will be made to keep information regarding my personal identity confidential.

In addition, I agree to maintain the confidentiality of other study participant information disclosed during any group discussions or anything observed during my participation in study activities, and to hold in confidence any and all study participant proceedings observed directly or indirectly.

By signing this consent form, I have not given up any of the legal rights that I have as a participant in a research study.

I agree to participate in this research study: **Yes** **No**

Participant printed name

____

Participant signature

____ **Date** ___________________

Researcher's statement

I, the undersigned, have fully explained the relevant details of this research study to the participant named above and believe that the participant has understood and has willingly and freely given their consent.

Researcher's printed name ___________________________ **Date** _________________

Signature ______________________________________

Role in the study ___________________________
