# Supplementary material for: Developing and testing a clinical care bundle incorporating caffeine citrate to manage apnoea of prematurity in a resource-constrained setting: a mixed methods clinical feasibility study protocol
Source: Implement Sci Commun. 2023 Jul 17;4:80. doi: 10.1186/s43058-023-00455-x (PMC10351121; doi:10.1186/s43058-023-00455-x)
Supplement: Supplementary file 4 — Additional file 4. REDCap tools. [file 43058_2023_455_MOESM4_ESM.pdf]

Screening and recruitment form

Study ID	______________________
Neonate IP Number:	______________________<br>(This is the internal hospital number for the patient)
What was the date of the eligibility assessment?	______________________<br>(Enter the date in DD-MM-YYYY format.)
What was the time of the eligibility assessment?	______________________<br>(Confirm that the time is entered correctly.)
REDCap username of the staff member using the screening tool:	______________________<br>(Confirm that your REDCap username/email address is shown correctly. If not, stop the screening, log out of REDCap and log in with your own REDCap username.)
What was the neonate's date of birth (DoB)?	______________________<br>(Enter the neonates DoB using the date using DD-MM-YYYY format.)
According to the neonate's admission chart, what was the gestational age of neonate at birth (in weeks)?	______________________<br>(Eligible neonates are under 34 weeks! Enter the gestational age of the neonate in weeks)
According to the neonate's admission chart, which of the following are available?	☐ U/S (Ultrasound)<br>☐ LMP (Last menstrual period)<br>☐ Neither U/S or LMP<br>☐ New Ballard Scoring<br>☐ None of the above<br>(If multiple gestation ages are present, select the gestational age that is more precise; ultrasound is most precise, followed by LNMP.)
The current chronological age of neonate (in days) is: calculated to be:	______________________
What was the birth weight of the neonate (in grams)?	______________________<br>(Enter the neonates birthweight, in grams, according to the chart.)
What was the source of the neonate's birth weight that was entered above?	◯ Labour ward chart<br>◯ NBU transfer form<br>◯ Other (specify): ______

Eligibility checklist

1. Was the neonate under 34 weeks gestational age at birth according to the LMP (last menstrual period) or U/S (ultrasound)?
- ◯ Yes
- ◯ No

(Note: Check neonatal chart)

1 If LMP or U/S is missing, was there a New Ballard Scoring for gestational assessment of fewer than 34 weeks?
- ◯ Yes
- ◯ No

2. Is the neonate currently receiving either aminophylline or caffeine citrate?
- ◯ Yes
- ◯ No

3. Has informed consent been obtained from the neonate's caregiver?
- ◯ Yes
- ◯ No

(Has the neonates caregiver provided and signed the informed consent document?)

Please STOP. Neonate is NOT eligible. If you think this is a mistake, review the answers above. DO NOT ENROLL UNTIL THIS ERROR DISAPPEARS.

Please STOP and obtain consent.DO NOT ENROLL UNTIL CONSENT IS OBTAINED.

Continue.Please enter the REDCap record ID on the Informed consent document before moving forward.

Enter the date and time that consent was given by the caregiver:

____

(What was the date and time that consent was provided?)

Eligibility assessment

The neonate is eligible for the study.Please proceed.

Eligibility confirmed by:

____

(Confirm that your REDCap username is shown correctly. If not, stop the screening, log out of REDCap and log in with your own REDCap username.)

I have assessed this child and the information contained on this screen accurately reflects the medical records of this child.
- ☐ Confirm

Time that eligibility was confirmed.

____

(Confirm the current time. Select the "NOW" button beside the displayed REDCap time to automatically update the time.)

Hospital admission data form

Study ID ________________________________

Mother's details

Mother first name: ______ First name of the mother as recorded on medical records. Do not enter abbreviations.
Mother age in years: ______ Mother's according to the admitting chart.
Estimated due date (EDD): ______ Enter the estimated due date on the admission chart.
Which of the following are available on the admission chart? ______
Gravida: ______ The number of times the mother has been pregnant, including all abortions, live and stillbirths. Note that this number includes the current pregnancy, therefore if this is the mother's first pregnancy enter '1'.
Parity: ______ Enter the parity according to the chart (eg: 2:1)
Was this a singleton pregnancy? ______
According to the antenatal and maternity charts, did the mother experience any notable health problems during this pregnancy? ______

Enter the date and time the New Ballard scoring was completed: ________________________________

What was the result of the New Ballard scoring? ________________________________
(Enter the result of the NBS)

According to the chart, what was the date of the last menstrual period (LMP)? ________________________________
(Enter from maternal chart or history the date of last menstrual period. in DD-MM-YYYY format)

According to the chart, were there any significant findings from ultrasound,?
- ◯ Yes
- ◯ No

Enter all the significant findings from the ultrasound, according to the chart: ________________________________
(Enter all detail included in chart.)

How many births from latest pregnancy?
- ◯ 2
- ◯ 3
- ◯ 4

(Total number of births in this pregnancy. For example triplets=3, twins=2. If fetal death occurs at or before 20 weeks, this is not counted under births this pregnancy.)

What is the birth order of the current neonate admitted to the study?
- ◯ 1
- ◯ 2
- ◯ 3
- ◯ 4

(Indicate the order in which the baby was born if it is part of a multiple birth. If fetal death has occurred at or before 20 weeks GA do not count in birth order.)

Please provide details of the notable health problems during this pregnancy:

____
(Enter as much detail as possible)

	Yes/Positive	No/Negative	Unknown
What is the PMTCT status of the mother?	◯	◯	◯
Is the mother currently on ARVs?	◯	◯	◯
Has the mother had either gestational diabetes or previous maternal diabetes (i.e. prior to conception)?	◯	◯	◯
Was maternal hypertension, preeclampsia or eclampsia present during pregnancy?	◯	◯	◯
Was there an "episode" of infection suspected or confirmed during the current pregnancy?	◯	◯	◯
Were antibiotics given during the pregnancy?	◯	◯	◯

Describe any other problems during this pregnancy as noted on the chart:

____
(Enter as much detail as required)

Relevant Family/Social History:

____
(Enter as much detail as required)

Labour and Birth

Membrane status:

- ☐ SROM
- ☐ ARM
- ☐ Known, but not noted on admission chart: SROM at home
- ☐ Known, but not noted on admission chart: SROM at another facility
- ☐ Known, but not noted on admission chart: SROM at KNH

(Note: Check newborn unit admission record)

Delivery (mode):

- ◯ SVD
- ◯ Vacuum
- ◯ Breech
- ◯ Elective CS
- ◯ Emergency CS

Please describe the reason for cesarian section in more detail:

____
(Enter as much detail as required)

Neonate's details from medical chart

Confirm neonate IP (Hospital) number: [ip_number] If IP number is incorrect, confirm you have the correct neonate file. If IP still does not match, review the screening form.
Date and time of birth: [neonate_dob]______ Date and time of birth according to obstetric and/or admitting records.
Date of admission to the newborn unit: ______ Date of admission to the newborn unit. This may be different than date of birth for late admissions or out-born babies.
Time of admission to the newborn unit: ______ Time of admission to the newborn unit.
Sex: ______ Record sex of neonate. If sex is listed as ambiguous or unknown, enter it as such.
Birth weight (grams): [neonate_birthweight] Confirm that the weight shown here is the same as the birth weight as recorded in the neonatal chart.
Head circumference (cm) at birth: ______ The first Occipito-Frontal Circumference (OFC) (Head Circumference) measured after admission, as noted in
the physician or nursing notes. Record in cm.
Length at birth (cm): ______ What was the neonate's recorded length at birth according to the chart?
Apgar score at 1 min: ______
Apgar score at 5 mins: ______
Apgar score at 10 mins: ______

Neonate admission assessment (taken from clinical chart)

Admission weight (in grams)? ______ Weight in grams as recorded at admission to the facility. When no admission weight is recorded, enter "Not present (NP)".
Neonates recorded temperature: (in degrees Celcius) on admission: ______ Body temperature in Celsius as recorded at admission to the facility. Record the first temperature listed
within five hours of admission. If the first recorded temperature is after 5 hours of admission enter "Not present (NP)"

What was the neonate's respiratory rate (in breaths per minute) at admission? ______ If no respiratory rate at admission is recorded, enter "Not present (NP)"
What was the neonate's pulse rate (in beats per minute) at admission: ______ If no pulse rate at admission is recorded, enter "Not present (NP)"
O2 saturation ______ If no O2 saturation at admission is recorded, enter "Not present (NP)"
Silverman-Anderson Score ______ If no Silverman Anderdon Score on the admission record, enter "Not present (NP)"

Where was the neonate transferred from?
- ◯ Theatre
- ◯ Labour ward
- ◯ Postnatal ward
- ◯ Pediatric ward
- ◯ Referral in from (specify): ______
- ◯ Home

Place of birth?
- ◯ Kenyatta National Hospital
- ◯ Home or roadside
- ◯ Other facility

Look at the labour and birth section on page 1 of the newborn unit admission record. Please copy the answers to the following information boxes.

	Yes	No
Meconium	◯	◯
Antenatal steroids?	◯	◯
BVM Resuscitation?	◯	◯
Cardiac compressions?	◯	◯
Oxygen?	◯	◯

CPAP?	◯	◯
Surfactant?	◯	◯
BCG?	◯	◯
TEO?	◯	◯
Vitamin K?	◯	◯
Chlorhexidine?	◯	◯

Look at the 'presenting problems and treatments given before admission' section on page 2 of the newborn unit admission record. Please copy the answers to the following information boxes.

	Yes	No
Fever	◯	◯
Difficulty in breathing	◯	◯
Difficulty in feeding	◯	◯
Convulsions/Twitching	◯	◯
Apnoea	◯	◯
Reduced/Absent movement	◯	◯
Urine in the past 12 hours	◯	◯
Passed meconium	◯	◯
Vomiting	◯	◯

Admission Diagnosis or Impression

Select all the admission diagnoses or impressions that are noted in the admission chart:

- ☐ Prematurity
- ☐ LBW (Low Birth Weight)
- ☐ Perinatal asphyxia
- ☐ RDS (Respiratory Distress Syndrome)
- ☐ Neonatal sepsis
- ☐ Meconium aspiration syndrome (MAS)
- ☐ Multiple gestation
- ☐ Meningitis
- ☐ Congenital anomaly
- ☐ Jaundice
- ☐ Other: ______

According to the admission record, was a random blood sugar done?

- ◯ Yes
- ◯ No

(Check Newborn Admission Record.)

Feeding during first few hours of admission

According to the neonatal monitoring chart, what type of feedings are listed within the first 6 hours of admission?

- ☐ Exclusively breast milk
- ☐ Exclusively formula
- ☐ Mix of breast milk and formula
- ☐ Baby not feeding
- ☐ Other
- ☐ None of the above

Was the first EBM (breast milk) feed within 4 hours of admission?

- ◯ Yes
- ◯ No

Enter the time of the first feed of EBM (breast milk): ____________________

Maternal meds at delivery

Study ID ______________________________

Please record all medications noted in the neonatal chart that the mother was taking.

Complete this form once for each medication. Repeat the form until all medications the mother is taking are entered.

Enter the name of the medication or treatment: ______ Hint: start writing the name of the medication and it will appear. If it does not appear, check the spelling. If it still does not appear, select [other] at the bottom of the list and enter the name of the medication in the next box.
Other medication: ______ If the medication name is not in the drop-down list above, please enter it here.

Are there additional medications that the mother is currently taking and have not been entered into the REDCap study database?
- ◯ Yes
- ◯ No

THIS FIELD IS HIDDEN TO RECRUITERS ______________________________

NOTE: Mark this form 'Unverified'. When done, please select 'Save and Add New Instance' below.Continue completing a new version of the Medications Treatment Log form until all current medications are entered.

Current neonate meds

Study ID

____

THIS FIELD IS HIDDEN TO RECRUITERS

____

Please record all medications the neonate is currently being given in this form.

Complete this whole form once for each medication. Repeat the form until all medications the neonate is taking are entered.

Enter the name of the medication or treatment: ______ Hint: start writing the name of the medication and it will appear. If it does not appear, check the spelling. If it still does not appear, select [other] at the bottom of the list and enter the name of the medication in the next box.
Other medication: ______ If the medication name is not in the drop-down list above, please enter it here.
What is the formulation of the [meds_name] given to the neonate? ______ Select the formulation of the [meds_name].

If the medication formulation is not in the list above, please enter it here.

What is the frequency (per day) of [meds_name] given? ______ Enter the frequency (per day) a dose of [meds_name] is given.

What is the dosage of [meds_name] given each time? ______ Enter the dose of the [meds_name] each time it is administered.
What is the route for the [meds_name]? ______ Select the route of the [meds_name].

If the route for the [meds_name] is not listed above, enter it here.

What day was the [meds_name] started? ______ Enter the first day of prescription in DD-MM-YYYY format.
Is the prescription of [meds_name] ongoing? ______
When was the [meds_name] stopped? ______ Enter the last day of prescription in DD-MM-YYYY format.
What is the duration (in days) for the [meds_name]? ______ Enter the duration, in days, that the meds have been prescribed.
Are there additional medications that the neonate is currently taking and have not been entered into the REDCap study database? ______

What is the total daily dose of [meds_name]?

____
(Enter as much detail as required.)

What is the indication for the [meds_name]?

____
(Enter as much detail as required.)

NOTE: Mark this form 'Unverified'. When done, please select 'Save and Add New Instance' below.Continue completing a new version of the Medications Treatment Log form until all current medications are entered.

Daily participant metrics

Study ID ______________________

This daily participant metrics form should be completed once per day the neonate is at the facility and be completed daily until the neonate has stopped receiving caffeine citrate or aminophylline for seven (7) days, or has been discharged from the hospital.

Enter the date of this data collection window: ______________________
(Confirm that the date entered correctly.)

Enter the start time of this data collection window: ______________________
(Confirm that the date and time are entered correctly.)

Does the current data collection window include a full 24 hours?
- ◯ Yes
- ◯ No

CONTINUE
Note: When answering all questions below, remember to only include the specific 24 hour time period starting at [metrics_start_time].

Why does the current data collection window not include a full 24 hours?
- ◯ Neonate was discharged
- ◯ It has not been 24 hours since the last time period was collected
- ◯ Other
- ◯ First day of admission

What time was the neonate admitted to the new-born unit (NBU)? ______________________

Enter the date and time the neonate was discharged ______________________
(This response will determine the end time for completing this daily metrics form.)

STOP
If it has not been 24 hours since the previous data collection window AND the neonate has not been discharged, discard this instrument and start again once it has been more than 24 hours after the previous data collection window.

Please describe the reason for completing the participant daily metrics form at an irregular time: ______________________
(Please provide as much detail as possible)

Breathing

How was the neonate breathing over the 24 hour time period?

- ◯ Breathing on own throughout the 24 hours
- ◯ Breathing with CPAP throughout the 24 hours
- ◯ Intubated throughout the 24 hours
- ◯ A combination of breathing on own and CPAP
- ◯ A combination of breathing on own and intubation
- ◯ A combination of CPAP and intubation
- ◯ A combination of breathing on own, CPAP and intubation

To the closest hour, rounding up, how many hours was the neonate on CPAP during the 24 hour time period?

____

To the closest hour, rounding up, how many hours was the neonate intubated during the 24 hour time period?

____

Location

What was the location of the neonate during the time period starting at [metrics_start_time]?

- ◯ Maternity ward
- ◯ Newborn unit

What room was the neonate in at [metrics_start_time]?

- ☐ Admission room
- ☐ Isolation room
- ☐ NICU 1
- ☐ NICU2/NHDU
- ☐ Preterm infant room B1
- ☐ Preterm infant room B2
- ☐ Nursery B3
- ☐ KMC room
- ☐ Other (specify): ______

During the current time window of interest, did the neonate receive any active warming?

Examples include:
Incubator
Rescuscitaire
Radiant warming
KMC

- ◯ Yes
- ◯ No

Select all of the warming methods used during the review time period:

- ☐ Incubator/rescuscitaire
- ☐ Radiant warmer
- ☐ KMC
- ☐ Other (specify): ______

(SELECT ALL THAT APPLY)

Neonate chart-based data

Current weight (in grams)?

____
(Enter the neonates current weight (in grams) at according to the monitoring chart for this day.)

Looking at the neonatal monitoring sheet for the 24 hour time period of interest, how many columns of vital signs are completed?	◯ 1<br>◯ 2<br>◯ 3<br>◯ 4<br>◯ 5<br>◯ 6<br>◯ 7<br>◯ 8<br>◯ 9<br>◯ 10<br>◯ 11<br>◯ 12 or more
Looking at the neonatal monitoring sheet for the 24 hour time period of interest, how many columns below the assessment section are completed?	◯ 1<br>◯ 2<br>◯ 3<br>◯ 4<br>◯ 5<br>◯ 6<br>◯ 7<br>◯ 8<br>◯ 9<br>◯ 10<br>◯ 11<br>◯ 12 or more

Vital signs

Heart Rate: Highest ______ Highest heart rate during the time period as recorded on the neonate's chart. If only one heart rate is recorded during the scoring period, enter this value as both the high and low value. If no heart rates were recorded, score as zero (0).
Heart Rate: Lowest ______ Lowest heart rate during the time period as recorded on the neonate's chart. If only one heart rate is recorded during the scoring period, enter this value as both the high and low value. If no heart rates were recorded, score as zero (0).
Respiratory Rate: Lowest ______ Lowest respiratory rate during the time period as recorded on the neonate's chart. If no respiratory rates were recorded, score as zero (0).
Respiratory Rate: Highest ______ Highest respiratory rate during the time period as recorded on the neonate's chart. If no respiratory rates were recorded, score as zero (0).
Temperature: Lowest ______ Lowest body temperature during the time period (axillary or rectal but not skin) as recorded on the neonate's chart, in Celsius.
SpO2: Lowest ______ Lowest SpO2 reading during the time period as recorded on the neonate's chart. If no SpO2 readings are recorded, score as zero (0).

Was feeding stopped during the 24 hour time period of note?	◯ Yes<br>◯ No
Which of the following new or ongoing conditions were noted on the patient's chart (doctor's continuation notes or cardex)?	☐ Severe lower chest wall indrawing<br>☐ Unconscious<br>☐ NEC (Necrotizing enterocolitis)<br>☐ Bowel distention<br>☐ Absent bowel sounds<br>☐ Other contraindication of enteral feeding<br>☐ None of the above<br>(Select all that apply)

Caffeine citrate & aminophylline perscriptions

Did the neonate have a new or ongoing caffeine citrate or aminophylline prescription during the 24 time period of interest?

- ◯ Yes, caffeine citrate only
- ◯ Yes, aminophylline
- ◯ Yes, both caffeine citrate and aminophylline have a new or ongoing prescription
- ◯ No, neither caffeine citrate nor aminophylline have a new or ongoing perscription

What was the reason for the caffeine citrate/aminophylline prescription?

- ◯ Prophylaxis
- ◯ Apnea
- ◯ Other (specify): ______

How many doses of caffeine citrate were administered during the time period of interest?

- ◯ 0 (None)
- ◯ 1 dose
- ◯ 2 doses
- ◯ 3 or more doses

First caffeine dose of the time period

What was the time of the first caffeine citrate dose on [metrics_start]? ______
What was the route of the first dose of caffeine citrate given during the current time period? ______
Was this caffeine citrate dose a loading or maintenance dose? ______

Second caffeine dose of the time period

What was the time of the second caffeine citrate dose on [metrics_start]? ______
What was the route of the second dose of caffeine citrate given during the current time period? ______
Was this second caffeine citrate dose a loading or maintenance dose? ______

How many doses of aminophylline were administered during the time period of interest?

____

Were caffeine citrate/aminophylline doses missed?

- ◯ Yes
- ◯ No
- ◯ Unknown.
- ◯ Unsure. The date is written but no signature is present.
- ◯ Unsure due to caffeine not being indicated AND not ticked on the treatment sheet.
- ◯ Not applicable, baby on 7-day follow-up

Why were doses missed?

- ◯ Unavailability of the drug/stockout
- ◯ Error
- ◯ Other (specify): ______
- ◯ Other (specify): ______

Were there caffeine citrate side effects noted on the chart during the 24 review time period?

- ◯ Yes
- ◯ No

What were caffeine citrate side effects noted on the chart?	☐ Death<br>☐ Tachycardia<br>☐ Tachypnea<br>☐ Agitation<br>☐ Irritability<br>☐ Tremor<br>☐ Serious intestinal disease with bloody stools (necrotising enterocolitis)<br>☐ Convulsion<br>☐ Allergic reaction<br>☐ Bloodstream infection (sepsis)<br>☐ Hyperglycaemia<br>☐ Arrhythmia<br>☐ Hypoglycaemia<br>☐ Failure to grow<br>☐ Feeding intolerance<br>☐ Brain injury<br>☐ Deafness<br>☐ Regurgitation<br>☐ Increase in stomach aspirate<br>☐ Increase in urine flow<br>☐ Increase in sodium and/or calcium in urine<br>☐ Reduced level of haemoglobin after prolonged treatment<br>☐ Reduced thyroid hormone at the start of treatment<br>☐ Other (specify): ______
Were there aminophylline side effects noted on the chart during the 24 review time period?	◯ Yes<br>◯ No
What were aminophylline side effects noted on the chart?	☐ Death<br>☐ Tachycardia<br>☐ Tachypnea<br>☐ Agitation<br>☐ Irritability<br>☐ Tremor<br>☐ Other (specify): ______
Was caffeine citrate/aminophylline terminated during the 24 hour time period of interest?	◯ Yes<br>◯ No
Enter time of termination:	______________________

Apnea recorded on the chart

Enter the number of apnea episodes recorded on the chart:	◯ 0<br>◯ 1<br>◯ 2<br>◯ 3<br>◯ 4<br>◯ 5<br>◯ 6<br>◯ 7<br>◯ 8<br>◯ 9<br>◯ More than 10

Select the estimated duration of the longest identified apnea episode during the 24 review time period:	◯ 20-45 seconds<br>◯ 46- 90 seconds<br>◯ 91-120 seconds<br>◯ longer than 121 seconds<br>◯ No data on apnea length available<br>(Duration as listed on patient chart only)
Was cyanosis/blue color identified on the chart during any of the apnea events during the 24-hour time window?	◯ Yes<br>◯ No
Was there bradycardia (HR < 60 bpm) recorded on the chart during the apnea episodes over the 24 review time period?	◯ Yes<br>◯ No
Enter number of bradycardia episodes recorded during apnea:	______________________
Estimated length of longest bradycardia episode:	______________________
Was there arrest episodes recorded during apnea?	◯ Yes<br>◯ No
Enter number of arrest episodes recorded during apnea:	______________________
Enter length of time (in seconds) of longest arrest episode:	______________________
Was there intubation/CPAP given during apnea?	◯ Yes<br>◯ No
Enter number of intubation (s)/CPAP intervention(s) recorded during apnea:	______________________
What were the causes of apnea noted on the chart during the 24 review time period?	☐ Sepsis<br>☐ Hypothermia<br>☐ Hypoglycemia<br>☐ Aspiration<br>☐ Seizure<br>☐ Apnea of prematurity<br>☐ Other: ______<br>☐ None
What is the lowest temperature recorded during the apnea-related hypothermia?	______________________
Was blood glucose measured during the apnea-related hypoglycemia?	◯ Yes<br>◯ No
What is the lowest blood glucose recorded during the apnea-related hypoglycemia?	______________________<br>(Enter lowest blood glucose in mmol/l)

Note the interventions performed during apnea within the 24 hours review time window	☐ Stimulation<br>☐ Oxygen<br>☐ CPAP<br>☐ Ventilation<br>☐ Repositioning<br>☐ BVM (Bag mask ventilation)<br>☐ Other: ______
Was a blood culture done after the apnea was identified?	○ No blood culture was done<br>○ Yes, a blood culture was done and the result was negative<br>○ Yes, a blood culture was done and the result was positive
Specify the number of times the stimulation was performed during apnea within the 24 hours review time window:	__________________________________
Specify the amount of oxygen provided during the 24 hour time period:	__________________________________
Specify the length of time the participant was on ventilation:	__________________________________

Desaturation recorded on chart

Was there desaturation (< 90%) recorded on the chart during the 24 review time period?	○ Yes<br>○ No
Which of the following desaturations were recorded on the chart during the 24 -hour review period?	☐ < 90%<br>☐ 91%-92%<br>☐ 93%-94%<br>☐ None of the above
What is the lowest saturation recorded on the chart during the 24 review time period?	__________________________________

Antibiotics

Was the neonate started on any new antibiotic during the 24-hour time window?	○ Yes<br>○ No

Do not forget to enter the details of the new antibiotic in the neonatal medication list.

Neonatal monitoring

Which of the following was the neonate attached to during the data collection window?	☐ Spot-check SpO2 monitor<br>☐ Continuous SpO2 monitor<br>☐ Continuous ECG monitor<br>☐ Rad-G monitor<br>☐ Draeger monitor in NICU<br>☐ None of the above<br>(Select all that apply)
Was the Rad-G device removed from the neonate during the time period of interest?	○ Yes<br>○ No

Why was the Rad-G continuous monitor removed from the neonate?

- ☐ Adverse event or reaction to Rad-G
- ☐ Rad-G moved to another neonate
- ☐ No longer indicated according to physician/patient chart
- ☐ Battery in Rad-G is empty
- ☐ Rad-G sensor malfunction
- ☐ Neonate discharged or exited study
- ☐ Other (specify): ______

(Select all that apply)

REMINDERPlease complete the "Rad-G reports" REDCap instrument as soon as you can.

Draeger monitor
Review the past 24 hours of data in the OxyCRG screen of the Draeger monitor. Enter the details below:

How many bradycardia events were logged in the OxyCRG review summary during the data collection window? ______ Bradycardia events are noted on the top-left-hand side of the OxyCRG review screen, labelled BRDY.
How many desaturation events were logged in the OxyCRG review summary during the data collection window? ______ Desaturation events are noted on the top of the OxyCRG review screen, labelled Desat.
How many apnea events were logged in the OxyCRG review summary during the data collection window? ______ Apnea events are noted on the top of the OxyCRG review screen, labelled APN.

Most recent apnea
Review the most recent apnea episode in the OxyCRG screen of the Draeger monitor. Enter the details below:

What was the time of the most recent apnea episode? ______
What was the highest heart rate shown during the most recent apnea episode? ______ Heart rate is shown on the right-hand side of the apnea review screen.
What was the lowest heart rate shown during the most recent apnea episode? ______ Heart rate is shown on the right-hand side of the apnea review screen.
What was the highest SpO2 shown during the most recent apnea episode? ______ SpO2 is shown on the right-hand side of the apnea review screen.
What was the lowest SpO2 shown during the most recent apnea episode? ______ SpO2 is shown on the right-hand side of the apnea review screen.
What was the length (in seconds) of the most recent apnea episode? ______ Apnea length (in seconds) is shown on the right-hand side of the apnea review screen.

Second most recent apnea
Review the second most recent apnea episode in the OxyCRG screen of the Draeger monitor. Enter the details below:

What was the time of the second most recent apnea episode? ______
What was the highest heart rate shown during the second most recent apnea episode? ______ Heart rate is shown on the right-hand side of the apnea review screen.
What was the lowest heart rate shown during the second most recent apnea episode? ______ Heart rate is shown on the right-hand side of the apnea review screen.
What was the highest SpO2 shown during the second most recent apnea episode? ______ SpO2 is shown on the right-hand side of the apnea review screen.
What was the lowest SpO2 shown during the second most recent apnea episode? ______ SpO2 is shown on the right-hand side of the apnea review screen.
What was the length (in seconds) of the second most recent apnea episode? ______ Apnea length (in seconds) is shown on the right-hand side of the apnea review screen.

Third most recent apnea

Review the third most recent apnea episode in the OxyCRG screen of the Draeger monitor. Enter the details below:

What was the time of the third most recent apnea episode? ______

What was the highest heart rate shown during the third most recent apnea episode? ______ Heart rate is shown on the right-hand side of the apnea review screen.

What was the lowest heart rate shown during the third most recent apnea episode? ______ Heart rate is shown on the right-hand side of the apnea review screen.

What was the highest SpO2 shown during the third most recent apnea episode? ______ SpO2 is shown on the right-hand side of the apnea review screen.

What was the lowest SpO2 shown during the third most recent apnea episode? ______ SpO2 is shown on the right-hand side of the apnea review screen.

What was the length (in seconds) of the third most recent apnea episode? ______ Apnea length (in seconds) is shown on the right-hand side of the apnea review screen.

SpO2 monitor

Enter the details below recorded on the continuous SpO2 monitor within the 24 hour time window:

How many alarm events AND apnea episodes were logged on the SpO2 monitor during the data collection window? ______

Enter the lowest oxygen saturation (SpO2) recorded by the monitor during the 24-hour time period: ______

Select the interventions that were performed during the apnea episode(s) identified on the SpO2 monitor: ______

Was there associated bradycardia with any of the SpO2 alarm or apnea events? ______

ECG Monitor

Enter the details below recorded on the ECG monitor within the 24 hour time window:

Enter the number of heart rate/pulse alarm events noted in the ECG: ______

Enter the number of respiratory rate alarm events: notes in the ECG:

______

What was the lowest heart rate/pulse recorded on the ECG in the current time window? ______

What was the lowest respiratory rate recorded on the ECG in the current time window? ______

Was there associated desaturation with any ECG alarms during the current time window? ______

End of participation form

Study ID	______________________
Has the neonate been discharged from the hospital?	◯ Yes<br>◯ No
What was the date and time that the neonate was discharged?	______________________
Why was the neonate discharged from the hospital?	☐ No reason<br>☐ 7-day follow-up complete<br>☐ Transfer to another facility<br>☐ Death<br>☐ Other (specify): ______
Was caffeine ongoing at discharge?	◯ Yes<br>◯ No
How many days was the neonate off caffiene as of [discharge_datetime]?	◯ 1<br>◯ 2<br>◯ 3<br>◯ 4<br>◯ 5<br>◯ 6<br>◯ 7<br>◯ More than 7
Upload the neonate's Rad-G monitoring data exported from Trace in CSV here:	(File should be in CSV format.)
Upload the neonate's Rad-G monitoring apnea report from Trace here:	(File should be in CSV format.)
