# Supplementary material for: Developing and testing a clinical care bundle incorporating caffeine citrate to manage apnoea of prematurity in a resource-constrained setting: a mixed methods clinical feasibility study protocol
Source: Implement Sci Commun. 2023 Jul 17;4:80. doi: 10.1186/s43058-023-00455-x (PMC10351121; doi:10.1186/s43058-023-00455-x)
Supplement: Supplementary file 5 — Additional file 5. ERC. [file 43058_2023_455_MOESM5_ESM.pdf]

UNIVERSITY OF NAIROBI
FACULTY OF HEALTH SCIENCES
P O BOX 19676 Code 00202
Telegrams: varsity
Tel:(254-020) 2726300 Ext 44355

KNH-UON ERC
Email: uonknh_erc@uonbi.ac.ke
Website: http://www.erc.uonbi.ac.ke
Facebook: https://www.facebook.com/uonknh.erc
Twitter: @UONKNH_ERC https://twitter.com/UONKNH_ERC

KENYATTA NATIONAL HOSPITAL
P O BOX 20723 Code 00202
Tel: 726300-9
Fax: 725272
Telegrams: MEDSUP, Nairobi

Ref: KNH-ERC/A/76

1st March, 2022

Prof. William M. Macharia
Principal Investigator-ETNA Study
Dept. of Paediatrics and Child Health
The Aga Khan University
P.O. Box 30270-00100
Nairobi, Kenya
Email Address: william.macharia@aku.edu

KENYATTA NATIONAL HOSPITAL
APPROVED
01 MAR 2022
QUALITY HEALTH CARE
KNH/UoN-ERC
P.O. Box 20723 - 00202, NAIROBI

Dear Prof. Macharia,

RESEARCH PROPOSAL: FEASIBILITY OF MANAGEMENT OF APNEA OF PREMATURITY WITH CAFFEINE CITRATE AT A TERTIARY HEALTH CARE FACILITY IN KENYA; A QUALITY IMPROVEMENT STUDY: A STUDY OF THE EVALUATION OF TECHNOLOGIES FOR NEONATES IN AFRICA (ETNA) PLATFORM-PROTOCOL VERSION 1.2 FEBRUARY 9, 2022 (P922/11/2021)

This is to inform you that KNH-UoN ERC has reviewed and approved your above research proposal. Your application tracking number is **P922/11/2021 and the approval letter ref. No is as indicated above.** The approval period is **1st March 2022 – 28th February 2023.**

In addition, it is noted that your request for waiver of consent to administer the study drug during the study period to enrolled participants has been adequately justified and is therefore approved.

This approval is subject to compliance with the following requirements;

i. Only approved documents including (informed consents, study instruments, MTA) will be used.
ii. All changes including (amendments, deviations, and violations) are submitted for review and approval by KNH-UoN ERC.
iii. Death and life threatening problems and serious adverse events or unexpected adverse events whether related or unrelated to the study must be reported to KNH-UoN ERC 72 hours of notification.
iv. Any changes, anticipated or otherwise that may increase the risks or affected safety or welfare of study participants and others or affect the integrity of the research must be reported to KNH-UoN ERC within 72 hours.
v. Clearance for export of biological specimens must be obtained from relevant institutions.

vi. Submission of a request for renewal of approval at least 60 days prior to expiry of the approval period. Attach a comprehensive progress report to support the renewal.

vii. Submission of an executive summary report within 90 days upon completion of the study to KNH-UoN ERC.

Prior to commencing your study, you will be expected to obtain a research license from National Commission for Science, Technology and Innovation (NACOSTI) https://research-portal.nacosti.go.ke and also obtain other clearances needed.

Yours sincerely,

01.03.2022

PROF. A.N. GUANTAI
CHAIR, KNH-UoN ERC

c.c. The Dean, Faculty of Health Sciences, UoN
The Senior Director, CS, KNH
The Assistant Director, Health Information, KNH
Co- Principal Investigators:
Prof. Grace Irimu, Dept of Paediatrics and Child Health, UoN
Dr. Amy Ginsburg, Infection Disease Physician & Epidemiologist, University of Washington, USA
Co-investigators:
Dr. Mary Waiyego, Consultant Neonatologist, KNH
Dr. Florence Murila, Neonatologist, Dept. of Paediatrics and Child Health, UoN
Dr. Rosaline Ochieng, Neonatologist, Aga Khan University, Nairobi
Dr. Jesse Coleman, Global Health Researcher, University of British Columbia, Canada
Dr. Ferdinard Okwaro, Medical Anthropologist, Aga Khan University, Nairobi
Dr. Jasmit Shah, Biostatistician/Bioinformatician, Aga Khan University Nairobi
Dr. Mark Ansermino, Paediatric Anaesthesiologist, University of British Columbia, Canada
