# Supplementary material for: Developing and testing a clinical care bundle incorporating caffeine citrate to manage apnoea of prematurity in a resource-constrained setting: a mixed methods clinical feasibility study protocol
Source: Implement Sci Commun. 2023 Jul 17;4:80. doi: 10.1186/s43058-023-00455-x (PMC10351121; doi:10.1186/s43058-023-00455-x)
Supplement: Supplementary file 6 — Additional file 6. ERC renewal. [file 43058_2023_455_MOESM6_ESM.pdf]

UNIVERSITY OF NAIROBI
FACULTY OF HEALTH SCIENCES
P O BOX 19676 Code 00202
Telegrams: varsity
Tel:(254-020) 2726300 Ext 44355

KNH-UON ERC
Email: uonknh_erc@uonbi.ac.ke
Website: http://www.erc.uonbi.ac.ke
Facebook: https://www.facebook.com/uonknh.erc
Twitter: @UONKNH_ERC https://twitter.com/UONKNH_ERC

KENYATTA NATIONAL HOSPITAL
P O BOX 20723 Code 00202
Tel: 726300-9
Fax: 725272
Telegrams: MEDSUP, Nairobi

Ref. No.KNH/ERC/R/20

2nd February, 2023

Prof. William M. Macharia
Principal Investigator- ETNA Study
Dept. of Paediatrics and Child Health
The Aga Khan University
P.O. Box 30270- 00100
Nairobi, Kenya
Email: william.macharia@aku.edu

Dear Prof. Macharia,

Re: Approval of Annual Renewal – Feasibility of management of apnea of prematurity with caffeine citrate at a tertiary healthcare facility in Kenya; A quality improvement study: A study of the evaluation of technologies for neonates in Africa (ETNA) platform (P922/11/2021)

Refer to your communication dated November 23, 2022.

This is to acknowledge receipt of the study progress report and hereby grant annual extension of approval for ethical research protocol P922/11/2021.

The approval dates are 1st March 2023 – 28th February 2024.

This approval is subject to compliance with the following requirements:

a) Only approved documents (informed consents, study instruments, advertising materials etc) will be used.
b) All changes (amendments, deviations, violations etc.) are submitted for review and approval by KNH- UoN ERC before implementation.
c) Death and life threatening problems and severe adverse events (SAEs) or unexpected adverse events whether related or unrelated to the study must be reported to the KNH- UoN ERC within 72 hours of notification.
d) Any changes, anticipated or otherwise that may increase the risks or affect safety or welfare of study participants and others or affect the integrity of the research must be reported to KNH- UoN ERC within 72 hours.
e) Submission of a request for renewal of approval at least 60 days prior to expiry of the approval period. (*Attach a comprehensive progress report to support the renewal*).
f) Clearance for export of biological specimens must be obtained from KNH- UoN-Ethics & Research Committee for each batch of shipment.
g) Submission of an executive *summary* report within 90 days upon completion of the study.

This information will form part of the data base that will be consulted in future when processing related research studies so as to minimize chances of study duplication and/or plagiarism.

Yours sincerely,

DR. BEATRICE K.M. AMUGUNE
SECRETARY, KNH- UoN ERC

cc. The Dean, Faculty of Health Sciences, UoN
The Senior Director, Clinical Services, KNH
The Chair, KNH-UoN ERC
